# Posicionamento sobre Indicações da Ecocardiografia em Cardiologia Fetal, Pediátrica e Cardiopatias Congênitas do Adulto – 2020

**DOI:** 10.36660/abc.20201122

**Published:** 2020-11-01

**Authors:** Samira Saady Morhy, Silvio Henrique Barberato, Alessandro Cavalcanti Lianza, Andressa Mussi Soares, Gabriela Nunes Leal, Ivan Romero Rivera, Marcia Ferreira Alves Barberato, Vitor Guerra, Zilma Verçosa de Sá Ribeiro, Ricardo Pignatelli, Carlos Eduardo Rochitte, Marcelo Luiz Campos Vieira

**Affiliations:** 1 Hospital Israelita Albert Einstein São PauloSP Brasil Hospital Israelita Albert Einstein, São Paulo, SP – Brasil; 2 Cardioeco Centro de Diagnóstico Cardiovascular CuritibaPR Brasil Cardioeco – Centro de Diagnóstico Cardiovascular, Curitiba, PR – Brasil; 3 Quanta Diagnóstico e Terapia CuritibaPR Brasil Quanta Diagnóstico e Terapia, Curitiba, PR – Brasil; 4 Universidade de São Paulo Faculdade de Medicina Instituto da Criança e do Adolescente São PauloSP Brasil Instituto da Criança e do Adolescente do Hospital das Clínicas da Faculdade de Medicina da Universidade de São Paulo (HC-FMUSP), São Paulo, SP – Brasil; 5 Hospital do Coração São PauloSP Brasil Hospital do Coração, São Paulo, SP – Brasil; 6 Hospital Evangélico de Cachoeiro de Itapemirim e Clínica CORImagem Cachoeiro de ItapemirimES Brasil Hospital Evangélico de Cachoeiro de Itapemirim e Clínica CORImagem, Cachoeiro de Itapemirim, ES – Brasil; 7 Hospital e Maternidade São Luiz Itaim São PauloSP Brasil Hospital e Maternidade São Luiz Itaim, São Paulo, SP – Brasil; 8 Santa Casa de Misericórdia MaceióAL Brasil Santa Casa de Misericórdia de Maceió, AL – Brasil; 9 The Hospital for Sick Children Toronto Canadá The Hospital for Sick Children, Toronto – Canadá; 10 Hospital Português SalvadorBA Brasil Hospital Português, Salvador, BA – Brasil; 11 Hospital Aliança SalvadorBA Brasil Hospital Aliança, Salvador, BA – Brasil; 12 Texas Children's Hospital Baylor College of Medicine HoustonTexas EUA Texas Children's Hospital, Baylor College of Medicine, Houston, Texas – EUA; 13 Universidade de São Paulo Faculdade de Medicina Instituto do Coração São PauloSP Brasil Instituto do Coração da Faculdade de Medicina da Universidade de São Paulo (InCor, FMUSP), São Paulo, SP – Brasil

**Realização:** Departamento de Imagem Cardiovascular (DIC) da Sociedade Brasileira de Cardiologia (SBC) e Sociedad de Imágenes Cardiovasculares de Sociedad Interamericana de Cardiología (Sisiac, Siac)

**Conselho de Normatizações e Diretrizes (2020-2021):** Brivaldo Markman Filho, Antonio Carlos Sobral Sousa, Aurora Felice Castro Issa, Bruno Ramos Nascimento, Harry Correa Filho, Marcelo Luiz Campos Vieira

**Coordenador de Normatizações e Diretrizes (2020-2021):** Brivaldo Markman Filho

**Editora Coordenadora:** Samira Saady Morhy

**Coeditores:** Silvio Henrique Barberato, Carlos Eduardo Rochitte, Marcelo Luiz Campos Vieira

**Table:** 

Declaração de potencial conflito de interesses dos autores/colaboradores do Posicionamento sobre Indicações da Ecocardiografia em Cardiologia Fetal, Pediátrica e Cardiopatias Congnitas do Adulto – 2020							
Se nos últimos 3 anos o autor/colaborador do Posicionamento:							
Nomes Integrantes do Posicionamento	Participou de estudos clínicos e/ou experimentais subvencionados pela indústria farmacêutica ou de equipamentos relacionados ao posicionamento em questão	Foi palestrante em eventos ou atividades patrocinadas pela indústria relacionados ao posicionamento em questão	Foi (é) membro do conselho consultivo ou diretivo da indústria farmacêutica ou de equipamentos	Participou de comitês normativos de estudos científicos patrocinados pela indústria	Recebeu auxílio pessoal ou institucional da indêstria	Elaborou textos científicos em periódicos patrocinados pela indústria	Tem ações da indústria
Alessandro Cavalcanti Lianza	Não	Não	Não	Não	Não	Não	Não
Andressa Mussi Soares	Não	Não	Não	Não	Não	Não	Não
Carlos Eduardo Rochitte	Não	Não	Não	Não	Não	Não	Não
Gabriela Nunes Leal	Não	Não	Não	Não	Não	Não	Não
Ivan Romero Rivera	Não	Não	Não	Não	Não	Não	Não
Marcelo Luiz Campos Vieira	Não	Não	Não	Não	Não	Não	Não
Marcia Ferreira Alves Barberato	Não	Não	Não	Não	Não	Não	Não
Ricardo Pignatelli	Não	Não	Não	Não	Não	Não	Não
Samira Saady Morhy	Não	Não	Não	Não	Não	Não	Não
Silvio Henrique Barberato	Não	Não	Não	Não	Não	Não	Não
Vitor C. Guerra	Não	Não	Não	Não	Não	Não	Não
Zilma Verçosa de Sá Ribeiro	Não	Não	Não	Não	Não	Não	Não

## 1. Introdução

De acordo com as “Normas para Elaboração de Diretrizes, Posicionamentos e Normatizações” sancionadas pela Sociedade Brasileira de Cardiologia, este documento foi escrito para atualizar as indicações da ecocardiografia em cardiologia fetal, pediátrica e cardiopatias congênitas do adulto, e complementa o posicionamento sobre indicações da ecocardiografia em adultos, recentemente publicado.1 Tal posicionamento não pretende ser uma ampla revisão da ecocardiografia em cardiopatias congênitas, mas sim um guia básico indispensável para amparar a tomada de decisão clínica racional do médico que solicita o exame. Embora leve em consideração os significativos avanços tecnológicos recentes da ecocardiografia, sua finalidade não é descrever com detalhes os métodos ecocardiográficos, mas resumir de forma clara e concisa as principais situações em que a ecocardiografia traz benefício para o diagnóstico e/ou a orientação terapêutica nesses grupos de pacientes. Optou-se, neste manuscrito, por destacar a classe da indicação (grau de recomendação), conforme a descrição a seguir:

– Classe I: condições para as quais há evidências conclusivas ou, na sua falta, consenso geral de que o exame é útil e seguro.
– Classe II: condições para as quais há evidências conflitantes e/ou divergência de opinião sobre utilidade e/ou segurança do exame.
– Classe IIa: evidências ou opiniões favoráveis ao exame. A maioria dos especialistas aprova.
– Classe IIb: utilidade e/ou segurança menos bem estabelecidas, havendo opiniões divergentes.
– Classe III: condições para as quais há evidências ou consenso de que o exame não é útil e, em alguns casos, pode ser prejudicial.

Em adição, foi descrito também o nível de evidência:

– A: diversos estudos clínicos randomizados concordantes ou metanálises robustas.
– B: dados de metanálises menos robustas ou estudo clínico randomizado único ou estudos observacionais.
– C: opinião de especialistas.

Assim, convencionou-se que, em todas as tabelas com recomendação do emprego da ecocardiografia nos diferentes cenários clínicos, constam as colunas com classe de indicação e nível de evidência

## 2. Ecocardiografia Fetal

A incidência da cardiopatia congênita é estimada entre 6-12/1.000 nascidos vivos;^2^^,^^3^ entretanto, estima-se que sua prevalência na vida fetal seja maior. Vários fatores estão associados ao aumento do risco de cardiopatia congênita em fetos, como os fatores familiares, condições maternas e fetais. A ecocardiografia fetal é a principal ferramenta para o diagnóstico detalhado das patologias cardíacas, desde o final do primeiro trimestre até o termo. O período para realização da ecocardiografia fetal é determinado por múltiplos fatores, tais como o motivo de sua indicação e a idade gestacional na qual a alteração cardíaca e/ou extracardíaca foi detectada. A ecocardiografia no rastreamento de gestação de risco deve ser feita a partir de 18 a 22 semanas de gestação. Lembrando que esse rastreamento inicial pode não identificar lesões evolutivas^4^ e arritmias.^5^^,^^6^ Portanto, achados anômalos na rotina obstétrica devem ser prontamente encaminhados para nova ecocardiografia fetal.

O ecocardiograma fetal pode ser realizado em idades gestacionais mais precoces, incluindo o final do primeiro e o início do segundo trimestre, geralmente em gestações de alto risco para cardiopatias congênitas, principalmente na presença de translucência nucal aumentada no ultrassom morfológico do primeiro trimestre.^7^^,^^8^ Ecocardiograma fetal transabdominal permite a visibilização adequada das estruturas cardíacas na maioria das gestações entre 13 a 14 semanas, permitindo detecção de anomalias. No entanto, o ecocardiograma transvaginal é necessário se a realização do exame for antes de 13 semanas, devido ao tamanho reduzido das estruturas cardíacas e da distância entre o feto e a parede abdominal da mãe.^7^^,^^8^ Quando o ecocardiograma fetal é realizado antes de 18 semanas, o exame deve ser repetido entre 18 a 22 semanas de gestação, devido à limitação de resolução de imagem, podendo não diagnosticar algumas lesões cardíacas, e também ao potencial de progressão de lesões não detectadas em idade gestacional precoce.^7^^–^^9^

O período e a frequência do ecocardiograma devem ser guiados por: gravidade da lesão, sinais de insuficiência cardíaca, mecanismos de progressão e avaliação para o manejo perinatal.

As recomendações para a ecocardiografia fetal estão listadas nas Tabelas 1 e 2.

**Tabela 1:** Recomendações para ecocardiografia fetal com perfil de gestação de risco elevado^5^^–^^9^

Recomendações	Grau de recomendação	Nível de evidência
DM pré-gestacional	I	A
DMG diagnosticada no primeiro trimestre	II	B
Fenilcetonúria maternal	I	A
Anticorpos maternos SSA/SSB	IIa	B
Medicacões maternas: Inibidores ECA Ácido retinoico AINH no terceiro trimestre	IIa I I	B B A
Infecção materna por rubéola no primeiro trimestre	I	C
Infecção materna com suspeita de miocardite/pericardite	I	C
Reprodução assistida	IIa	A
Cardiopatia congênita em parente de primeiro grau	I	B
Cardiopatia com herança mendeliana em parente de primeiro ou segundo grau	I	C
Suspeita de cardiopatia no ultrassom obstétrico	I	B
Anomalia extracardíaca fetal	I	B
Feto com alteração cromossômica	I	C
Feto com taquicardia ou bradicardia ou batimentos irregulares frequentes	I	C
TN > 95%	I	A
Gemelaridade monocoriônica	I	A
Feto com hidropisia ou derrames	I	B

AINH: anti-inflamatórios não hormonais; DM: diabetes melito; DMG: diabetes melito gestacional; ECA: enzima conversora de angiotensina; TN: transluscência nucal. Adaptada de Donafrio et al.^7^

**Tabela 2:** Recomendações para ecocardiografia fetal com perfil de gestação de risco baixo^5^^–^^9^

Recomendações	Grau de recomendação	Nível de evidência
Medicações maternas: Anticonvulsivante Lítio Vitamina A Inibidores de serotonina AINH no primeiro e segundo trimestre	IIb	B
Cardiopatias em parentes de segundo grau	IIb	B
Alterações do cordão umbilical e placenta	IIb	C
Alteração venosa intra-abdominal fetal	IIb	C

AINH: anti-inflamatórios não hormonais. Adaptada de Donafrio et al.^7^

## 3. Ecocardiografia no Recém-nascido

Os recém-nascidos deixam uma situação de circulação em paralelo com baixa resistência vascular sistêmica e alta resistência vascular pulmonar na vida fetal para uma circulação em série, em que o débito cardíaco de ambos os ventrículos deve ser igual, na presença de uma alta resistência vascular sistêmica. Essas mudanças circulatórias que ocorrem após o nascimento podem demorar de dias a semanas para se completarem, principalmente nos prematuros, por causa de uma inabilidade de as comunicações presentes na vida fetal se fecharem prontamente. Assim, a persistência do canal arterial (PCA), a persistência de altas pressões pulmonares e a incapacidade do miocárdio imaturo de bombear sangue contra uma resistência vascular sistêmica, repentinamente alta, podem levar a uma redução transitória do fluxo sanguíneo sistêmico e alterar a hemodinâmica desses pacientes.^9^ Além disso, a presença de anomalias cardíacas estruturais ou de alterações extracardíacas, como sepse ou hérnia diafragmática, é tolerada de forma diferente nessa faixa etária.^10^

A fisiologia transicional da circulação cardiovascular no período neonatal faz com que esses pacientes precisem ser avaliados como um grupo à parte.

As razões mais comuns para realização do ecocardiograma no período neonatal são para reconhecer ou excluir doenças cardíacas congênitas estruturais em pacientes com: sopro cardíaco, alteração do teste do coraçãozinho,^11^ pacientes em choque, hipoxêmicos, em insuficiência respiratória ou com múltiplas malformações. Seguidas pelas anomalias funcionais, como avaliação da persistência do canal arterial, da hemodinâmica pulmonar e da função cardíaca (ver Tabela 2).

A avaliação ecocardiográfica de pacientes internados em unidades de terapia intensiva neonatal se justifica, inclusive de forma evolutiva, como um fator de mudanças específicas no manejo clínico do neonato.

As recomendações para a ecocardiografia em recém-nascidos estão listadas na Tabela 3.

**Tabela 3:** Recomendações para ecocardiografia em recém-nascidos^9^^,^^11^^–^^15^

Recomendações	Grau de recomendação	Nível de evidência
Sopro cardíaco patológico ou outras anormalidades na ausculta cardíaca	I	C
Cianose central, insuficiência cardíaca, choque cardiogênico, desconforto respiratório	I	A
Assimetria de pulsos e/ou gradiente de pressão arterial entre membros superiores e inferiores	I	A
Cardiomegalia ao exame radiológico de tórax ou encontros anormais sugestivos de doença cardíaca	I	A
Síndromes associadas à doença cardiovascular	I	B
Anomalias extracardíacas	I	B
Anomalia da posição cardíaca ou do situs	I	B
Ecocardiograma fetal e/ou obstétrico alterado ou duvidoso para malformação cardíaca	I	C
Cirurgia cardíaca corretiva ou paliativa	I	B
História de hidropisia fetal	I	B
Suspeita clínica de canal arterial patente	I	A
Avaliação do significado hemodinâmico do PCA, seguimento dos efeitos da terapêutica	I	A
Avaliação evolutiva do neonato submetido à cirurgia para fechamento de canal arterial com instabilidade hemodinâmica	I	A
Asfixia perinatal com alteração hemodinâmica e/ou alteração de biomarcadores	I	A
Suspeita de hipertensão pulmonar	I	A
Avaliação evolutiva da hipertensão pulmonar em tratamento medicamentoso	I	A
Hipotensão	I	A
Avaliação da posição das cânulas do suporte de vida extracorpóreo, manutenção e desmame da ECMO	I	A
Doença sistêmica materna associada à conhecida anomalia neonatal	IIa	B
Infecção materna durante a gestação ou parto com potencial sequela cardíaca fetal ou neonatal	IIa	B
Diabetes materna sem ecocardiograma fetal ou com ecocardiograma fetal normal	IIb	B
Fenilcetonúria maternal	I	A
Disfunção autoimune materna	IIa	B
Exposição materna a teratógenos	IIa	B
Dificuldade de crescimento na ausência de anormalidades clínicas definidas	IIa	C
História de ritmo cardíaco ectópico fetal não sustentado, na ausência de arritmia após o parto	III	C
Acrocianose com saturação normal pelo oxímetro de pulso nas extremidades superiores e inferiores	III	C
Avaliação morfológica e funcional no pós-operatório de cirurgia cardíaca	I	B
Avaliar derrame pericárdico e impacto hemodinâmico e guiar procedimentos intervencionistas	I	A
Identificar posição do cateter venoso central e suas complicações (trombose e infecção)	I	A

ECMO: oxigenação por membrana extracorpórea; PCA: persistência do canal arterial.

## 4. Ecocardiografia em Lactentes, Crianças e Adolescentes

Por ser um método não invasivo e prover informações anatômicas, hemodinâmicas e fisiológicas dos corações pediátricos, o ecocardiograma é o principal método diagnóstico para avaliação inicial das cardiopatias congênitas ou adquiridas em lactentes, crianças e adolescentes.

Crianças com doenças cardíacas representam um grupo variado de pacientes, frequentemente caracterizado por malformação anatômica complexa, que necessitam de seguimento durante toda a vida. Assim, estudos seriados podem ser indicados para monitoramento de função valvar, crescimento de estruturas cardiovasculares, função ventricular e seguimento de intervenções medicamentosas ou cirúrgicas.^9^^,^^16^^–^^18^

Sinais e sintomas como cianose, déficit de crescimento, dor torácica induzida por exercício, síncope, desconforto respiratório, sopros, insuficiência cardíaca, alteração de pulsos e cardiomegalia podem sugerir doença cardíaca estrutural.

Além disso, o ecocardiograma pode ser indicado mesmo sem quadro clínico específico em pacientes com história familiar de doença cardíaca hereditária, síndromes genéticas associadas à cardiopatia estrutural, exames complementares alterados (ecocardiografia fetal, radiografia de tórax e eletrocardiograma).

Pacientes com arritmias podem ter cardiopatia estrutural, como transposição corrigida das grandes artérias e anomalia de Ebstein, tumores cardíacos e cardiomiopatias. As arritmias sustentadas e o uso de medicações antiarrítmicas podem levar à alteração na função miocárdica, sendo importante a realização do ecocardiograma no manejo clínico desses pacientes.

As recomendações para a ecocardiografia em lactentes, crianças e adolescentes estão listadas na Tabela 4.

**Tabela 4:** Recomendações para ecocardiografia em lactentes, crianças e adolescentes^9^^,^^12^^,^^16^^–^^18^

Recomendações	Grau de recomendação	Nível de evidência
Sopro patológico ou outra evidência de anormalidade cardíaca	I	C
Anomalia da posição cardíaca ou do situs	I	B
Cardiomegalia ao exame radiológico de tórax ou encontros anormais sugestivos de doença cardíaca	I	B
Alteração ao eletrocardiograma	I	B
Avaliação pré-operatória imediata de cirurgia cardíaca	I	C
Mudança no quadro clínico de paciente com cardiopatia conhecida	I	B
Avaliação morfológica e funcional no pós-operatório de cirurgia cardíaca	I	C
História familiar de doença cardíaca transmitida geneticamente	I	B
Doença neuromuscular com envolvimento miocárdico	I	B
Sinais e sintomas de endocardite infecciosa	I	A
Sinais e sintomas de insuficiência cardíaca	I	A
Palpitações sem outros sintomas, história familiar benigna e eletrocardiograma normal	IIb	C
Palpitações com história familiar de arritmia, morte súbita, cardiomiopatia.	I	B
Palpitações em paciente com cardiomiopatia conhecida	I	B
Palpitações com eletrocardiograma anormal ou canulopatia conhecida	IIa	C
Assimetria de pulsos periféricos	I	A
Síndrome associada com doença cardiovascular; genótipo positivo para cardiomiopatia; anomalia cromossômica associada à doença cardiovascular	I	B
Determinação do momento adequado para tratamento clínico ou cirúrgico em pacientes com cardiopatia conhecida	I	B
Seleção, implantação, patência e monitoramento de dispositivos endovasculares.	I	A
Identificação de shunts intracardíacos e intravasculares antes, durante e após cateterismo cardíaco intervencionista percutâneo	I	A
Febre prolongada, sem causa aparente, em paciente com cardiopatia congênita	I	A
Sopro funcional em paciente assintomático	IIb	C
Retardo do crescimento na ausência de anormalidade clínica definida	IIb	C
Dor torácica atípica, identificada como origem musculoesquelética em paciente assintomático	III	
Síncope com eletrocardiograma anormal, síncope ao exercício	I	A
Síncope com história familiar de cardiomiopatia ou morte súbita	I	A
Síncope neurocardiogênica (vasovagal)	IIa	C
Dor torácica ao esforço ou dor torácica em repouso com eletrocardiograma anormal	I	B
Dor torácica associada à febre ou uso de drogas ilícitas	IIa	B
Sopro inocente presumível com sinais e sintomas de doença cardíaca	I	C
Cianose central	I	A
Deformidade da parede torácica e escoliose pré-operatória	IIb	C
Início, manutenção e desmame do suporte de vida extracorpóreo	I	B
Ecocardiograma normal prévio com mudança no estado cardiovascular e/ou nova história familiar sugestiva de doença cardíaca hereditária	IIa	C
Biomarcadores cardíacos anormais	I	B
Hemoglobinopatias	I	B
Doenças do tecido conectivo (Marfan, Loeys, Dietz e outras)	I	B
Distrofia muscular	I	B
Doenças autoimunes	I	B
Hipertensão arterial	I	A
Acidente vascular encefálico	I	B
Doença metabólica, mitocondrial ou de depósito	I	B
História familiar de doença cardiovascular: morte súbita antes dos 50 anos de idade, doenças do tecido conectivo (Marfan ou síndrome de Loeys Dietz), hipertensão arterial idiopática	IIa	C
História familiar de doença cardiovascular: cardiomiopatia hipertrófica, cardiomiopatia dilatada não isquêmica, hipertensão arterial pulmonar hereditária	IIa	B

## 5. Ecocardiografia Pediátrica nas Cardiopatias Adquiridas

As cardiopatias adquiridas ocorrem principalmente em doenças sistêmicas associadas a processos inflamatórios, doenças renais, uso de quimioterápicos cardiotóxicos, doença parenquimatosa pulmonar e após transplante cardíaco.

O acometimento miocárdico pode ocorrer em várias condições, tais como doenças inflamatórias sistêmicas (principalmente naquelas com curso mais agressivo como lúpus eritematoso sistêmico juvenil, artrite idiopática juvenil e febre reumática).^19^^–^^22^ Durante a realização de quimioterapia cardiotóxica (principalmente os antracíclicos) e radioterapia em região mediastinal, o ecocardiograma está indicado antes, durante e após o tratamento, com intuito de indicar medidas cardioprotetoras e até mesmo mudar a terapêutica em alguns casos.^23^

Nos pacientes nefropatas crônicos/hipertensos e/ou dialíticos, o ecocardiograma fornece informações preciosas ao clínico no que diz respeito à geometria ventricular, função sistólica/diastólica e volemia. Isso, muitas vezes, orienta a mudança do esquema de diálise, assim como introdução ou troca de anti-hipertensivos e de fármacos vasoativos.^24^

Nos pacientes pneumopatas, o ecocardiograma viabiliza não apenas a estimativa de pressões pulmonares, mas também a avaliação do desempenho ventricular direito, que guarda importante correlação com o prognóstico clínico.^25^^–^^27^

Em crianças e adolescentes com AIDS, o ecocardiograma é utilizado para investigação de acometimento cardíaco direto pelo vírus, que podem causar miocardiopatia dilatada, hipertensão pulmonar e até hipertrofia ventricular, além dos efeitos de doenças oportunistas e/ou efeitos colaterais das medicações utilizadas.^28^

Com o número crescente de insuficiência cardíaca final em crianças, faz-se necessário avaliação pré e pós-transplante cardíaco e/ou cardiopulmonar^29^ além de auxiliar na tomada de decisão para introducão/retirada de suporte cardiovascular.^30^

As recomendações para a ecocardiografia em recém-nascidos, lactentes, crianças e adolescentes com cardiopatia adquirida estão listadas na Tabela 5.

**Tabela 5:** Recomendações para ecocardiografia em recém-nascidos, lactentes, crianças e adolescentes com cardiopatia adquirida^9^^,^^16^^–^^31^

Recomendações	Grau de recomendação	Nível de evidência
Avaliação inicial e reavaliações de pacientes com diagnóstico suspeito ou confirmado de síndrome de Kawasaki, arterite de Takayasu, miopericardites, AIDS e febre reumática	I	B
Pós-transplante cardíaco ou cardiopulmonar	I	B
Avaliação inicial e reavaliações de pacientes em uso de quimioterápicos cardiotóxicos e com radioterapia mediastinal	I	B
Avaliação inicial e reavaliações em pacientes com doença miocárdica	I	C
Doença renal grave e/ou hipertensão arterial sistêmica para avaliação de acometimento cardíaco	I	B
Avaliação de doadores para transplante cardíaco	I	C
Hipertensão arterial pulmonar	I	A
Avaliação evolutiva da hipertensão arterial pulmonar em terapia medicamentosa ou cirúrgica	I	B
Início ou suspensão de suporte cardiopulmonar extracorpóreo	I	C
Evento tromboembólico	I	C
Sepse, insuficiência cardíaca direita ou cianose em paciente com cateter venoso	I	B
Embolização sistêmica ou pulmonar em paciente com fluxo direita-esquerda e cateter venoso	I	C
Síndrome de veia cava superior em paciente com cateter venoso	I	C
Doença hepática	IIa	C
Obesidade com outros fatores de risco cardiovasculares ou apneia obstrutiva do sono	IIa	C
Sepse	IIa	B
Fibrose cística sem evidência de *cor pulmonale*	IIa	C
Acompanhamento de pacientes após febre reumática sem evidência de envolvimento cardíaco	IIb	C
Avaliação cardíaca após pericardite sem evidências de pericardite recorrente ou pericardite crônica	IIb	C
Febre em paciente com cateter venoso sem evidências de embolização sistêmica ou pulmonar	IIb	C
Avaliação de rotina para participação em esportes competitivos em pacientes com exame cardiovascular normal	IIb	C
Acompanhamento tardio de síndrome de Kawasaki sem evidências de anormalidades coronarianas na fase aguda	III	C
Avaliação de rotina em paciente assintomático com cateter venoso	III	C

## 6. Ecocardiografia em Adultos com Cardiopatia Congênita

A cardiologia pediátrica experimentou notáveis avanços nos últimos 30 anos, tanto no aspecto diagnóstico com o advento da ecocardiografia quanto no tratamento de correção das cardiopatias, inicialmente cirúrgico e posteriormente por técnica percutânea na sala de hemodinâmica. Dados recentes indicam que, em 2010, a população estimada de adultos com cardiopatia congênita nos EUA era de 1,4 milhão de pacientes^30^ Esta população apresenta problemas relacionados com defeitos residuais, novos defeitos adquiridos, como o refluxo pulmonar após correção definitiva de tetralogia de Fallot, ou obstruções após cirurgia de Jatene, arritmias, insuficiência cardíaca, doença adquirida do adulto, endocardite infecciosa ou indicação de transplante cardíaco. Muitos sobrevivem com cirurgias paliativas que podem ou não precisar de correção definitiva, como Senning, Mustard, Rastelli, Glenn ou Fontan, que trazem novas complicações implícitas no método cirúrgico adotado e, ainda, muitos pacientes apresentam-se pela primeira vez, sem diagnóstico prévio da cardiopatia.^32^^–^^35^

Não há dúvida de que a ecocardiografia transtorácica bidimensional tem papel importante no diagnóstico e no acompanhamento dessas malformações.^36^ Avanços recentes como a ecocardiografia 3D mostraram superioridade na determinação de volumes e até mesmo da função ventricular, principalmente em malformações complexas como aquelas que apresentam fisiologia univentricular ou na avaliação do ventrículo direito, devendo ser empregadas sempre que houver disponibilidade e pessoal treinado para o seu uso.^37^ Ainda, a orientação cirúrgica da imagem em 3D permite melhor compreensão quando apresentadas ao cirurgião, o que possibilita um melhor planejamento cirúrgico. Da mesma forma, novas técnicas para a avaliação da função diastólica e da função segmentar como Doppler tecidual, *strain* ou *strain rate* podem ser de grande utilidade, principalmente nas condições com fisiologia univentricular ou naquelas com deformação das câmaras cardíacas, principalmente do ventrículo direito^38^ (ver nos itens 9 e 10).

A principal limitação da ecocardiografia na avaliação de adultos com cardiopatia congênita é a inadequada janela transtorácica em pacientes com cirurgia cardíaca prévia ou deformidades da parede torácica, assim como a ecocardiografia não é adequada na avaliação do arco aórtico, das artérias coronárias, das artérias pulmonares e dos vasos colaterais. Nessas situações, a ecocardiografia transesofágica, angiotomografia e a ressonância magnética (RM) são extremamente úteis.

As recomendações para a ecocardiografia em adultos com cardiopatias congênitas estão listadas na Tabela 6.

**Tabela 6:** Recomendações para a ecocardiografia em adultos com cardiopatias congênitas^9^^,^^29^^,^^36^^,^^38^^–^^44^

Recomendações	Grau de recomendação	Nível de evidência
Avaliação estrutural e funcional inicial na suspeita de cardiopatia congênita evidenciada por sopro, cianose, insaturação arterial, anormalidade ao eletrocardiograma ou radiografia de tórax	I	C
Mudança no quadro clínico em paciente com cardiopatia congênita conhecida, operada ou não	I	C
Dúvidas do diagnóstico original ou anormalidades estruturais ou hemodinâmicas não esclarecidas em paciente com cardiopatia congênita conhecida	I	C
Acompanhamento de pacientes com comunicação interventricular para avaliação de modificações morfológicas evolutivas	I	C
Acompanhamento periódico de pacientes com cardiopatia congênita, operada ou não, nos quais é necessária a avaliação da função contrátil, valvar e de condutos	I	C
Acompanhamento anual em pacientes no pós-operatório de correção total, parcial ou paliativa, com defeitos residuais e sequelas que possam comprometer a evolução clínica do paciente	I	C
Identificação da origem e curso inicial das artérias coronárias	I	C
Avaliação da síncope pós-exercício não explicada para definição diagnóstica inicial	I	C
Avaliação de lesão aórtica em pacientes com suspeita ou confirmação de síndrome de Marfan para avaliação seriada da aorta e/ou da valva mitral	I	C
Exames periódicos em pacientes operados de PCA, CIA, CIV, coarctação aórtica ou valva aórtica bicúspide, sem defeito residual e sem mudanças na condição clínica	III	C
Acompanhamento de pacientes com cardiopatias sem significado hemodinâmico e sem mudança na condição clínica	III	C
Avaliação de lesões do arco aórtico, artérias pulmonares e colaterais, cuja anatomia é melhor definida por outros métodos de diagnóstico	III	C
Avaliação periódica de malformação cardíaca sem alteração no exame físico, na condição clínica do paciente ou em outros exames como eletrocardiograma e radiografia de tórax	III	C
CIA: comunicação interatrial; CIV: comunicação interventricular; PCA: persistência do canal arterial.	III	C

## 7. Ecocardiografia Transesofágica em Cardiologia Pediátrica

O ecocardiograma transesofágico (ETE) constitui uma via de acesso alternativa, por meio do uso de transdutores especiais, que fornece maior definição das estruturas cardíacas, aumentando as possibilidades diagnósticas do método.

É particularmente importante na definição de estruturas anatômicas complexas e anormalidades funcionais, nem sempre passíveis de avaliação pela ecocardiografia transtorácica isolada.

Com os avanços tecnológicos e miniaturização das sondas, a adoção do ETE vem se ampliando no campo da cardiologia pediátrica, podendo oferecer importantes informações para os pacientes desde a faixa etária neonatal até adolescentes e adultos, tanto no diagnóstico, na avaliação intraoperatória, no pós-operatório imediato e tardio e na unidade de terapia intensiva quanto no laboratório de hemodinâmica, auxiliando a realização de procedimentos intervencionistas.

### 7.1. Ecocardiografia Transesofágica como Ferramenta Diagnóstica

O ETE deve ser adotado para melhor definição diagnóstica da cardiopatia, nas situações em que é necessária melhor avaliação anatômica em algumas cardiopatias congênitas específicas, na maioria das vezes em adultos, visto que a qualidade da imagem em crianças é geralmente boa ao exame transtorácico (Tabela 7).

**Tabela 7:** Recomendações para a ecocardiografia transesofágica como ferramenta diagnóstica^9^^,^^45^

Recomendações	Grau de recomendação	Nível de evidência
Confirmação ou exclusão de uma suspeita diagnóstica clínica relevante não demonstrada pelo ETT	I	A
Informações anatômicas e hemodinâmicas insuficientes pelo ETE, principalmente em crianças com deformidades torácicas ou com obesidade e em adultos com cardiopatias congênitas	I	A
Avaliação de forame oval patente (FOP) como possível etiologia de eventos embólicos centrais ou periféricos em pacientes jovens (< 60 anos), com contraste salino agitado para determinar a possibilidade de fluxo direita-esquerda. Avaliar fatores de risco do FOP para AVE/AIT*: aneurisma de septo interatrial, passagem de um número > 30 microbolhas do átrio direito para o átrio esquerdo, túnel do FOP > 10 mm e valva de Eustáquio proeminente	I	A
Na avaliação de forame oval patente pré-implante de marca-passo transvenoso	I	A
Classificação, dimensão e localização do defeito septal atrial, principalmente em pacientes adultos e naqueles com definição transtorácica inadequada para seleção de possíveis candidatos à oclusão percutânea e escolha da prótese.	I	A
Avaliação de dissecção da aorta nas síndromes de Marfan, Ehlers-Danlos, Turner e na coartação de aorta	I	A
Avaliação da aorta na arterite de Takayasu	I	A
Avaliação dos tubos intra ou extracardíacos no pós-operatório de cirurgia de Senning, Mustard ou Fontan	I	A
Na avaliação de trombos, massas, vegetações, abscessos e próteses	I	A
Na determinação do grau e mecanismos de refluxo valvar mitral para auxiliar reparo cirúrgico ou percutâneo (Mitraclip)	I	B

*AVE/AIT: acidente vascular encefálico/ataque isquêmico transitório; ETE: ecocardiograma transesofágico; ETT: ecocardiograma transtorácico.*

### 7.2. Ecocardiografia Transesofágica no Intraoperatório

O grande impacto da ecocardiografia transesofágica na sala cirúrgica é a detecção de defeitos residuais importantes que muitas vezes não são suspeitados. Vários autores têm demonstrado retorno à circulação extracorpórea para revisão cirúrgica após a realização do ETE intraoperatório com taxas variando de 6 a 11,4% dos casos, nas diferentes séries avaliadas.^46^

As indicações de ETE intraoperatório para cardiopatias congênitas estão listadas na Tabela 8.

**Tabela 8:** Recomendações para a ecocardiografia transesofágica no intraoperatório^9^^,^^45^^–^^46^

Recomendação	Grau de recomendação	Nível de evidência
Avaliação perioperatória da anatomia e da função cardíaca	I	A
Monitoramento de procedimentos cirúrgicos com riscos de fluxos anormais, refluxos valvares, obstruções residuais ou disfunção miocárdica ventricular	I	A
Cirurgias minimamente invasivas, guiadas por vídeo e procedimentos híbridos	I	A

### 7.3. Ecocardiografia Transesofágica na Unidade de Terapia Intensiva (UTI)

A definição das imagens pelo ETT no pós-operatório imediato pode estar prejudicada pela presença de drenos, curativos, telas e ventilação mecânica, sendo necessária a adoção do ETE, que pode fornecer informações anatômicas (lesões residuais) e hemodinâmicas importantes na condução clínica e na terapêutica dos pacientes (Tabela 9).

**Tabela 9:** Recomendações para a ecocardiografia transesofágica na UTI^9^^,^^45^

Recomendação	Grau de recomendação	Nível de evidência
Avaliação de defeitos residuais, derrame pericárdico, função ventricular em pacientes com má qualidade de janela transtorácica	I	A
Monitoramento pós-operatório em paciente com esterno aberto	I	A

### 7.4. Ecocardiografia Transesofágica no Laboratório de Hemodinâmica

O ETE auxilia a intervenção hemodinâmica detalhando o diagnóstico em diversas cardiopatias, monitorando o procedimento, além de fornecer informações anatômicas do resultado e de eventuais lesões residuais^47^ (Tabela 10).

**Tabela 10:** Recomendações para a ecocardiografia transesofágica no laboratório de hemodinâmica^9^^,^^45^^,^^47^

Recomendação	Grau de recomendação	Nível de evidência
No fechamento percutâneo de forame oval patente, comunicações interatriais e interventriculares	I	A
No fechamento de fenestrações em pós-operatório de cirurgia de Fontan	I	A
Nas dilatações das tunelizações das cirurgias de Senning e de Mustard	I	A
Nas estenoses de artérias pulmonares e de tubos com colocação de stents	IIb	B
Orientação na valvoplastia mitral e reparo valvar mitral percutâneo (Mitraclip)	I	A
Orientação nas valvoplastias pulmonar e aórtica	IIa	A
Implante de endopróteses aórticas para tratamento de aneurismas, dissecções, hematomas ou úlceras parietais da aorta torácica	I	A
Orientação do cateter na perfuração e dilatação percutânea de valvas atrésicas	I	A
Durante cateterismo intervencionista terapêutico para ablação por radiofrequência	I	A

## 8. Ecocardiografia sob Estresse em Cardiologia Pediátrica

A ecocardiografia sob estresse (físico ou farmacológico) é uma técnica bem estabelecida em adultos.^48^^,^^49^ Na faixa etária pediátrica, não temos ainda uma diretriz ou recomendação específica; no entanto, como na população adulta, a aplicação em crianças e adolescentes tem sido mais concentrada na pesquisa de doença isquêmica,^50^^–^^56^ mas está em expansão para outras áreas de aplicação não necessariamente isquêmica^55^^–^^63^ (Tabela 7).

Ambos os tipos de estresse, farmacológico e exercício, podem ser aplicados em crianças, com algumas peculiaridades.^64^^–^^66^ A dobutamina é o agente farmacológico mais comum e usado nos mesmos protocolos dos pacientes adultos. Em geral, em crianças abaixo de 8 anos de idade, recomenda-se sedação ou até mesmo anestesia. O exercício físico pode ser usado em crianças acima de 8 anos de idade que são cooperativas e com habilidades para se exercitar, em esteira ou bicicleta.^67^

## 9. Ecocardiografia Tridimensional

A ecocardiografia tridimensional (3D) tem sido incorporada na prática clínica, adicionando informações ao ecocardiograma bidimensional (2D), principalmente para a abordagem dos defeitos congênitos nos quais a visão tridimensional oferece uma visão muito próxima dos planos anatômicos e cirúrgicos.^68^ Esse mesmo conceito se aplica aos procedimentos feitos no laboratório de hemodinâmica, nos quais a visão tridimensional pode não só guiar os procedimentos, mas também avaliar de melhor forma a anatomia para adequar aos dispositivos usados. Avaliação dos volumes ventriculares e função também tem sido alvo da tecnologia 3D, principalmente por avaliar a geometria ventricular nas mais diversas formas nos defeitos congênitos, incluindo os corações univentriculares.^69^^,^^70^ A avaliação das valvas atrioventriculares é possível não só do ponto de vista do detalhamento anatômico, incluindo o aparelho subvalvar, como também a avaliação funcional da movimentação do anel valvar, interação entre o movimento dos folhetos valvares e cordas.^71^

Quando se trata de paciente pediátrico, temos uma grande vantagem pela melhor janela acústica transtorácica, e, mais recentemente, houve um avanço no desenvolvimento de transdutores com um *foot print* menor e com frequência mais alta (2 a 8 Mhz). Entretanto, ainda não temos a mesma qualidade de imagem quando se usa a combinação 2D-3D com o mesmo transdutor, principalmente em pacientes pequenos. Outro grande desafio ainda a ser ultrapassado é o desenvolvimento do transdutor transesofágico pediátrico, o que limita o uso do ETE 3D em pacientes acima de 30 kg de acordo com as recomendações dos fabricantes. Em crianças pequenas, recomenda-se o uso de transdutores pediátricos com frequência mais alta, e também o ecocardiograma epicárdico, nos casos realizados no intraoperatório. O ecocardiograma 3D transesofágico deve ser sempre considerado em pacientes maiores (geralmente acima de 30 kg) se o 3D transtorácico não oferecer informação suficiente para o planejamento cirúrgico ou intervenção.

Existe uma variedade de defeitos congênitos nos quais o ecocardiograma 3D pode adicionar informações sobre as mais diversas estruturas anatômicas, incluindo septos atrial e ventricular, as valvas semilunares e atrioventriculares, assim como as vias de saída. Como tem havido um progresso tecnológico, a aplicação está se estendendo à medida que ocorre esse progresso e adequação para população pediátrica. Atualmente, o uso baseia-se mais na necessidade clínica de informações adicionais do que em estudos randomizados sobre a vantagem do 3D sobre o 2D. Portanto, é individualizado e de acordo com o perfil do laboratório de imagem ou hospital adotar a tecnologia para lesões específicas.

As lesões valvares e os defeitos septais isolados são as principais indicações; contudo, em situações em que há concomitante anomalia de conexão ventriculoarterial, como nas duplas vias de saída do ventrículo direito, a posição e o tamanho da comunicação interventricular podem ser melhor visualizados e demonstrados pelo ecocardiograma 3D.

De acordo com a área ou estrutura avaliada pelo ecocardiograma 3D transtorácico e/ou transesofágico, podemos ter informações relevantes que complementariam aquelas obtidas pelo ecocardiograma 2D.^72^^–^^83^ As artérias pulmonares, a valva pulmonar e até mesmo a via de saída do ventrículo direito, assim como o arco aórtico, adicionam pouca informação quando avaliadas pelo 3D (Tabela 12).

**Tabela 11:** Recomendações para ecocardiografia sob estresse em cardiologia pediátrica

Recomendação	Grau de recomendação	Nível de evidência
Pesquisa de insuficiência coronária em crianças pós-transplante cardíaco tardio	IIa	B
Avaliação tardia na doença de Kawasaki com alterações coronarianas na fase aguda	IIa	B
Pós-operatório de cirurgia de Jatene, pós-operatório de origem e trajetos anormais das artérias coronárias e fístulas coronário-cavitárias	IIa	B
Função ventricular nas miocardiopatias e nas insuficiências valvares mitral e aórtica	IIa	B
Rastreamento de disfunção ventricular em pacientes que recebem quimioterapia com antraciclinas e no pós-transplante, a fim de avaliar função miocárdica durante o exercício	IIa	B
Pesquisa de insuficiência coronária em crianças com atresia pulmonar com septo ventricular íntegro, dislipidemia, diabetes melito insulinodependente, estenose aórtica supravalvar	IIb	B
Avaliação do comportamento do gradiente de pressão em cardiomiopatia hipertrófica, estenoses valvares pulmonar e aórtica	IIb	B
Avaliação da reserva miocárdica em pós-operatório tardio de cirurgias em plano atrial para transposição dos grandes vasos, avaliação do ventrículo direito em pós-operatório tardio de tetralogia de Fallot	IIb	B

**Tabela 12:** Informações adicionais do ecocardiograma 3D sobre estruturas anatômicas específicas e recomendações^72^^–^^78^^,^^80^^–^^82^^,^^87^^,^^88^^,^^91^

Estrutura anatômica de interesse	Modalidade	Informações adicionais	Grau de recomendação	Nível de evidência
Septo interatrial	ETT/ETE	Dimensão, formato, localização do(s) defeito(s)	I – Defeitos complexos ou residuais II – Defeito central e único	B B
Valva tricúspide	ETT/ETE	Morfologia dos folhetos, aparelho subvalvar (cordas), localização dos jatos regurgitantes	I	B
Valva mitral	ETT/ETE	Morfologia dos folhetos, aparelho subvalvar (cordas), localização dos jatos regurgitantes	I	B
Septo interventricular	ETT/ETE	Dimensão, formato, localização do(s) defeito(s) complexo (s)	I	B
Via de saída do VE	ETT/ETE	Morfologia da obstrução subaórtica	I	B
Valva aórtica	ETT/ETE	Medida da valva aórtica, morfologia dos folhetos, mecanismo de regurgitação	I	B
Via de saída do VD	ETT/ETE	Morfologia e visualização do local de obstrução	III	C
Valva pulmonar	ETT	Morfologia	IIa	C

*ETT: ecocardiograma transtorácico; ETE: ecocardiograma transesofágico; VD: ventrículo direito; VE: ventrículo esquerdo.*

O ecocardiograma 3D também adiciona informação no contexto de algumas cardiopatias congênitas específicas em que existem anomalias de conexão (atrioventricular ou ventriculoarterial)^76^^,^^84^^–^^86^ (Tabela 13).

**Tabela 13:** Informações adicionais do ecocardiograma 3D em defeitos congênitos e recomendações^71^^,^^79^^,^^83^^–^^86^

Cardiopatia congênita	Modalidades	Informações adicionais	Grau de recomendação	Nível de evidência
DSAV	ETT/ETE	Dimensão do defeito atrial e/ou ventricular; morfologia dos folhetos e do aparelho subvalvar; avaliação do(s) jato(s) regurgitante(s); dimensões dos orifícios e ventrículos nos defeitos desbalanceados	I	B
Conexão AV discordante	ETT/ETE	Morfologia e função das valvas tricúspide e mitral, localização e dimensão da CIV associada; morfologia das vias de saída do VD e VE	I	B
TGA complexas	ETT/ETE	Morfologia e função das valvas tricúspide e mitral, localização e dimensão da CIV, anatomia das vias de saída do VD e VE nos casos de obstrução	I	B
Tetralogia de Fallot	ETT	Dimensão e localização da CIV e anatomia da via de saída do VD	III	C
Truncus Arteriosus	ETT/ETE	Morfologia da valva truncal*	III	C
Dupla via de saída do VD	ETT	Relação das valvas atrioventriculares, da posição e do tamanho da CIV com as grandes artérias	III	C

*AV: atrioventricular; CIV: comunicação interventricular; DSAV: defeito do septo atrioventricular; ETT: ecocardiograma transtorácico; ETE: ecocardiograma transesofágico; TGA: transposição das grandes artérias.*

* Especificamente para avaliação da valva truncal em pacientes mais velhos.

A aplicação do ecocardiograma 3D na sala de hemodinâmica para fechamento de defeitos dos septos atrial e ventricular complementa as imagens 2D para delimitar as bordas dos defeitos e estruturas relacionadas,^87^^,^^88^ especificamente nas comunicações atriais tipo *Ostium Secundum*, que são muito bem demonstradas por imagem em tempo real pelo ecocardiograma 3D transesofágico. O fechamento das comunicações interventriculares por dispositivos percutâneos ou transmural também pode ser guiado pelo ecocardiograma 3D, principalmente para avaliar estruturas próximas, como, por exemplo, folhetos e/ou cordas da valva tricúspide. Existem outras aplicações na sala de hemodinâmica no qual o ecocardiograma 3D pode guiar os procedimentos: fechamento das fenestrações na cirurgia de Fontan, fistulas coronárias, rupturas do seio de Valsalva, regurgitação paravalvar, perfuração de septo e localização dos eletrodos no processo de ressincronização ventricular.^89^^–^^94^

Um dos grandes desafios em cardiopatias congênitas é a avaliação dos volumes e função ventricular, por razões intrínsecas dos defeitos congênitos (posição cardíaca, anomalias de conexão, material não contrátil e diferenças de pré-carga ventricular, entre outras). Os *softwares* disponíveis foram desenvolvidos a partir da geometria ventricular esquerda de corações normais, o que muitas vezes invalida as informações obtidas pelo 3D. Apesar de as medidas de volumes e fração de ejeção serem replicáveis, o ecocardiograma 3D tem mostrado valores menores que a RM na quantificação dos volumes, o que impede de substituir um pelo outro. Portanto, a aplicação clínica ainda tem sido dificultada por ausência de valores de normalidade na população pediátrica. Não se recomenda o uso dos *softwares* desenvolvidos para ventrículo esquerdo ou direito normais em ventrículos congenitamente malformados até que novos *softwares* ou modelos tenham sido validados.*70,95-97*

A recomendação geral para o uso do ecocardiograma 3D transtorácico em pediatria é que deve ser a de acordo com o tipo de paciente e com o perfil do laboratório de ecocardiograma e/ou hospital.

Existe o consenso de que o 3D é uma modalidade que complementa o ecocardiograma 2D, e não uma substituição, independentemente do tipo de lesão.

10. Estudo da Deformação Miocárdica em Pacientes Pediátricos

A deformação miocárdica (*strain*) vem se mostrando ferramenta útil na avaliação da função diastólica e sistólica, tanto em adultos como na população pediátrica.^98^ O estudo do *strain* miocárdico pelo *speckle-tracking* é um método independente do ângulo de insonação e apresenta baixa variação intra e interobservador, o que permite quantificar a função ventricular global e regional de forma mais acurada do que os métodos tradicionais, como Doppler tecidual, fração de encurtamento ou de ejeção.^99^ Alguns estudos já demonstraram elevado valor prognóstico do *strain* obtido pelo *speckle-tracking*, reforçando sua utilidade tanto em patologias congênitas como adquiridas.^100^

No entanto, o *strain* miocárdico está sujeito a variações fisiológicas causadas por idade, sexo, frequência cardíaca, pré-carga, pressão arterial e superfície corpórea, além do tipo de *software* utilizado para análise.^101^ Um esforço contínuo vem sendo feito no sentido de estabelecer valores normais de *strain* que possam ser utilizados como referência universal em pediatria, para que a avaliação da deformação miocárdica seja incorporada aos *guidelines* e comece a ser adotada na rotina clínica.^102^^–^^104^ Por ora, o estudo da deformação miocárdica nas diversas doenças pediátricas tem grau de recomendação II e nível de evidência B.

### 10.1. *Strain* Ventricular em Cardiopatias Adquiridas na Infância

A avaliação do *strain* ventricular direito e esquerdo é particularmente útil em situações nas quais se pretende identificar disfunção sistólica e/ou diastólica em fase subclínica. As informações obtidas através da análise do *strain* possibilitam intervenção terapêutica oportuna em diversas doenças sistêmicas que cursam com acometimento miocárdico.

A detecção precoce de lesão miocárdica secundária ao uso de antracíclicos é uma das mais relevantes contribuições do estudo da deformação miocárdica até o momento, já tendo sido incorporada em protocolos de acompanhamento de pacientes oncológicos.^105^^–^^108^

Já foi demonstrada a correlação entre o grau de atividade inflamatória e os valores de *strain e de strain rate* sistólico e diastólico do VE em pacientes com doenças reumatológicas, como lúpus eritematoso sistêmico juvenil.^20^

Outros trabalhos comprovaram a eficácia do *strain* obtido pela técnica de *speckle-tracking* na detecção de miocardite, não só de etiologia autoimune como também viral.^109^^,^^110^ O padrão de comprometimento regional do *strain* de VE, nos casos de miocardiopatia dilatada em crianças, influencia a evolução para óbito ou para transplante, como demonstrado por Forsha et al.,^111^ Outra utilidade do *strain* nos casos de miocardiopatia dilatada é detectar dissincronia, identificando os casos que podem se beneficiar de terapia de ressincronização.^111^

O estudo com *strain* pós-transplante cardíaco ortotópico em crianças pode identificar, com razoável sensibilidade e especificidade, os indivíduos que irão manifestar doença vascular do enxerto nos anos subsequentes.^112^ Alguns relatos, incluindo pequeno número de crianças transplantadas, sugere associação entre a redução de *strain* segmentar e a presença de rejeição em biópsias endomiocárdicas, o que favorece a técnica como instrumento diagnóstico menos invasivo em um futuro próximo.^113^^–^^115^

Em pacientes jovens com distrofia muscular de Duchenne, estudos demonstraram redução significativa do *strain* longitudinal e radial das paredes inferolateral e anterolateral do VE, mesmo antes do comprometimento da fração de ejeção ou do surgimento de sintomas de insuficiência cardíaca.^116^ Vários trabalhos têm demonstrado melhora do desempenho cardiovascular e da sobrevida em 10 anos dos pacientes com Duchenne que passam a receber inibidores da enzima de conversão da angiotensina e betabloqueadores já aos primeiros sinais ecocardiográficos de deterioração miocárdica, quando ainda são assintomáticos do ponto de vista cardiovascular.^117^

O estudo do *strain* miocárdico também tem contribuído para detecção de comprometimento miocárdico em doenças de depósito, como as mucopolissacaridoses (MPS)^118^ e a doença de Pompe.^119^ Trabalhos têm centrado atenção no *strain* miocárdico como parâmetro de avaliação do impacto da reposição enzimática a longo prazo sobre a função ventricular dos portadores dessas doenças.^120^

A análise do *strain* miocárdico também surge como uma possibilidade de diagnóstico precoce de inflamação miocárdica e disfunção ventricular nos casos de doença de Kawasaki.^51^ McCandless et al.^121^ evidenciaram redução do *strain* longitudinal de VE ao ecocardiograma inicial de pacientes com Kawasaki, que posteriormente vieram a desenvolver dilatação coronariana ou que mostraram resistência ao tratamento com imunoglobulina. Esses achados sugerem que o *strain* de VE pode vir a ser utilizado em breve como ferramenta de estratificação de risco em Kawasaki.^121^

Nos casos de disfunção miocárdica induzida por sepse pediátrica, o *strain* longitudinal e circunferencial do VE parecem já estar reduzidos em fases iniciais, a despeito da fração de ejeção ainda conservada.^122^

Em pacientes adultos com insuficiência renal crônica (IRC), já foi comprovada a redução do *strain* longitudinal do VE, mesmo em estágios iniciais da doença e ainda com fração de ejeção preservada. Atribui-se o comprometimento precoce da deformação miocárdica à fibrose induzida por inflamação crônica e por toxinas urêmicas. Além disso, a disfunção endotelial que acompanha a IRC pode acarretar resposta vasodilatadora inadequada, causando isquemia em um ventrículo já hipertrófico. Achados semelhantes já foram documentados também em populações pediátricas, restando estabelecer se essa redução do *strain* longitudinal do VE pode ser utilizada como um preditor específico de morbidade e mortalidade em crianças com IRC.^123^

Alterações cardiovasculares são comuns em indivíduos infectados pelo HIV, mas frequentemente são subdiagnosticadas e não tratadas, o que impacta na qualidade de vida e na mortalidade a longo prazo. São atribuídas tanto ao efeito direto do vírus quanto às medicações antirretrovirais sobre o miocárdio e a vasculatura. A disfunção sistólica sintomática é normalmente encontrada apenas nos casos mais avançados da síndrome de imunodeficiência adquirida.^124^ Trabalhos mais recentes realizados em crianças e adultos jovens comprovaram comprometimento do *strain* longitudinal de VD e VE, ainda em pacientes assintomáticos e com fração de ejeção do VE normal. Diante desses resultados, Naami et al. sugeriram, em 2016, a incorporação do estudo da deformação miocárdica aos ecocardiogramas dos pacientes pediátricos com HIV, com o objetivo de identificar pacientes com disfunção subclínica e com maior risco cardiovascular.^125^

Em um estudo que incluiu adolescentes e adultos jovens com talassemia submetidos a múltiplas transfusões, Chen et al.^126^ identificaram correlação negativa entre a ferritina sérica e o *strain* longitudinal do VE. Além disso, mesmo após correção para sexo, idade, nível de ferritina sérica e índice de massa ventricular, o *strain* longitudinal de VE permaneceu como preditor independente de eventos adversos em pacientes talassêmicos, como insuficiência cardíaca, arritmias e óbito (HR: 6,05; *p* = 0,033).^127^

Investigando crianças e adolescentes portadores de hipertensão pulmonar idiopática (HPI), Okumura et al. comprovaram o valor prognóstico da avaliação seriada do *strain* longitudinal do VD na população pediátrica. Um valor de *strain* inferior a –14% ao ecocardiograma inicial identificou pacientes que evoluíram para transplante pulmonar ou óbito com 100% de sensibilidade e 54,5% de especificidade. Concluíram que a deformação miocárdica na HPI pediátrica é ferramenta mais sensível do que os parâmetros convencionais de avaliação da função do VD (TAPSE – do inglês *tricuspid annular plane systolic excursion*, FAC – do inglês *fractional area change*, velocidade da onda S tricúspide) para a detecção de pacientes com pior prognóstico.^127^ Em publicação recente, Hooper et al.^128^ comprovaram a utilidade do *strain* longitudinal de VD no seguimento clínico da HPI em crianças, demonstrando que os valores de *strain* apresentam excelente correlação com os valores de BNP – do inglês *B-type natriuretic peptide values*, no decorrer do tratamento com análogos da prostaciclina.^13^ A Tabela 14 apresenta graus de recomendação e níveis de evidência.

**Tabela 14:** Recomendações para o strain ventricular em cardiopatias adquiridas na infância^20^^,^^51^^,^^105^^–^^128^

Indicação	Grau de recomendação	Nível de evidência
Cardiotoxicidade em oncopediatria	IIa	B
Miocardites: autoimunes e virais	IIa	B
Miocardiopatia dilatada: seleção para terapia de ressincronização	IIa	B
Doença vascular do enxerto pós-TX cardíaco	IIb	B
Rejeição pós-TX cardíaco	IIb	B
Distrofias musculares (p. ex., Duchenne)	IIa	B
Doenças de depósito (p. ex., Pompe e MPS)	IIa	B
Doença de Kawasaki	IIa	C
Sepse	IIb	B
Insuficiência renal crônica	IIb	B
Infecção pelo HIV/AIDS	IIa	B
Anemias crônicas (p. ex., talassemia)	IIa	B
Hipertensão pulmonar	IIa	B

AIDS: síndrome de imunodeficiência adquirida; HIV: human immunodeficiency virus; MPS: mucopolissacaridoses; TX: transplante.

### 10.2. *Strain* Ventricular em Cardiopatias Congênitas

A análise do *strain* longitudinal do VD em posição subpulmonar provou-se factível e reprodutível na avaliação perioperatória de diferentes cardiopatias congênitas.^129^ Entretanto, na presença de obstrução residual significativa no pós-operatório (PO), parâmetros de avaliação da função sistólica longitudinal do VD, tais como TAPSE, velocidade da onda S e *strain* de pico sistólico longitudinal, não apresentam adequada correlação com a fração de ejeção obtida pela RM. Em situações com estenose pulmonar residual ou uma combinação de estenose e insuficiência pulmonar, a hipertrofia do VD acarreta predomínio de fibras circunferenciais, alterando o padrão de deformação dessa câmara, que habitualmente depende mais das fibras longitudinais.^130^ Hayabuchi et al.^131^ avaliaram o *strain* de pico sistólico circunferencial da parede livre do VD ao corte subcostal, especificamente crianças portadoras de cardiopatias congênitas com sobrecarga pressórica ao VD. Dessa forma, encontraram melhor correlação entre os valores de *strain* e de fração de ejeção de VD.^131^ Trabalhos envolvendo crianças assintomáticas em pós-operatório tardio de tetralogia de Fallot (T4F) identificaram comprometimento do *strain* de pico sistólico longitudinal biventricular. Alguns autores encontraram correlação negativa entre o *strain* de pico sistólico longitudinal de VD e fração de ejeção do VD e fração de regurgitação pulmonar, ambas estimadas pela RM.^132^ Outros trabalhos documentaram correlação negativa entre o *strain* longitudinal do VE e o grau de regurgitação pulmonar, reforçando a importância da interdependência entre os ventrículos.^133^ Ainda que o estudo da deformação miocárdica consiga detectar disfunção sistólica subclínica nos pacientes operados de T4F que evoluem com regurgitação pulmonar, infelizmente, não há consenso quanto a um valor de corte de *strain* que permita indicar o melhor momento para a troca valvar pulmonar.

O VD em posição sistêmica também demonstra mudança do padrão de deformação miocárdica, com predomínio de contração das fibras circunferenciais. A redução discreta do *strain* longitudinal nesta condição representa uma alteração na geometria ventricular direita e não disfunção sistólica real. Trata-se de um mecanismo adaptativo, que faz com que a contratilidade do VD sistêmico se torne semelhante à contratilidade do VE. Por essa razão, trabalhos recentes sugerem uma faixa de valores normais de *strain* de pico sistólico longitudinal do VD sistêmico que ficam abaixo do esperado para o VD subpulmonar (–10% a –14,5%).^130^ Já valores de *strain* longitudinal de VD inferiores a –10% foram associados à ocorrência de eventos adversos, em PO tardio de cirurgia de Senning.^134^

A seleção de pacientes com ventrículo único (VU) para cirurgia de Fontan leva em consideração a resistência vascular pulmonar e a pressão diastólica final ventricular. No entanto, os critérios atuais de indicação mostram-se falhos para uma considerável parcela desses pacientes, que acabam enfrentando complicações e internações prolongadas. Quando associado à resistência vascular pulmonar e à pressão diastólica final ventricular, o *strain rate* circunferencial pré-operatório melhora a estratificação de risco para pacientes com VU candidatos à cirurgia de Fontan, não importando se o ventrículo é de morfologia direita ou esquerda.^135^

No caso da anomalia de Ebstein, o estudo da deformação miocárdica acrescenta pouco à avaliação da função ventricular direita, uma vez que o *strain* mostra fraca correlação com a fração de ejeção obtida pela RM.^136^

Castaldi et al.^137^ demonstraram utilidade do *strain* longitudinal do ventrículo esquerdo no diagnóstico de pacientes com obstrução coronariana em PO tardio de correção de origem anômala de coronária esquerda. Um valor de *strain* < –14,8% ao ecocardiograma identificou segmentos miocárdicos com fibrose à RM, com sensibilidade de 92,5% e especificidade de 93,7%.^137^

Dusenbery et al.^138^ reforçaram essa associação entre redução do *strain* longitudinal do VE e a presença de fibrose miocárdica, avaliando crianças e adultos jovens com estenose valvar aórtica e com fração de ejeção do VE preservada.^138^ É sabido que adultos com estenose aórtica que apresentam realce tardio à RM com gadolínio e valores reduzidos de *strain* longitudinal do VE têm maiores taxas de mortalidade após intervenção valvar.^138^ Ver Tabela 15 para graus de recomendação e níveis de evidência.

**Tabela 15:** Recomendações para o strain ventricular em cardiopatias congênitas^129^^–^^135^^,^^137^

Indicação	Grau de recomendação	Nível de evidência
Avaliação da função do VD subpulmonar (p. ex., T4F)	IIb	B
Avaliação da função do VD sistêmico (p. ex., PO de cirurgia de Senning, TCGA)	IIb	B
Avaliação do VU pré-cirurgia de Fontan	IIb	B
Avaliação do VU pós-cirurgia de Fontan	IIb	B
Avaliação de VE após correção de OACE	IIa	B
Avaliação da função de VE em estenose aórtica	IIb	B

OACE: origem anômala da coronária esquerda; PO: pós-operatório; T4F: tetralogia de Fallot; TCGA: transposição corrigida das grandes artérias; VD: ventrículo direito; VE: ventrículo esquerdo; VU: ventrículo único.

### 10.3. *Strain* Atrial Direito e Esquerdo em Pediatria

O estudo da mecânica atrial direita através do *speckle-tracking* foi incorporado recentemente à pediatria, surgindo como ferramenta promissora para a detecção de disfunção ventricular direita. Hope et al.^139^ encontraram redução significativa do *strain* longitudinal de átrio direito em crianças com HPI. O *strain* atrial mostrou-se mais sensível e específico do que parâmetros convencionais de avaliação da função ventricular direita na identificação dos pacientes com HPI que vieram a apresentar desfechos desfavoráveis (óbito, transplante pulmonar e/ou cardíaco).^139^

Diversos trabalhos têm descrito as implicações clínicas da medida do *strain* atrial esquerdo pela técnica de *speckle-tracking*. O *strain* de átrio esquerdo, na fase de reservatório, mostrou-se mais acurado na estimativa da pressão diastólica final do VE do que parâmetros ecocardiográficos clássicos como o volume atrial esquerdo e a relação E/E', além de correlacionar-se inversamente com os níveis plasmáticos do NT-ProBNP.^140^

### 10.4. Perspectivas de Utilização do Strain Ventricular no Feto

Trabalhos recentes têm sugerido que a análise da deformação miocárdica pode contribuir para a avaliação da função sistólica e diastólica biventricular também nos fetos. Como exemplo, Miranda et al. documentaram redução de *strain* rate diastólico precoce e tardio, no eixo longitudinal de VD e VE, em fetos de mão diabética. Além disso, registraram redução do strain de pico sistólico longitudinal do ventrículo direito, mediante comparação com fetos normais de mesma idade gestacional. Os autores ressaltam que o comprometimento da deformação diastólica ocorreu independentemente da presença de hipertrofia septal. Concluem que o estudo da deformação miocárdica pode detectar alterações subclínicas nos fetos de mãe diabética, antes que os parâmetros ecocardiográficos clássicos sejam capazes de fazê-lo.^141^
