# Supplementary material for: Posicionamento sobre Indicações da Ecocardiografia em Cardiologia Fetal, Pediátrica e Cardiopatias Congênitas do Adulto – 2020
Source: Arq Bras Cardiol. 2020 Nov 1;115(5):987–1005. [Article in Portuguese] doi: 10.36660/abc.20201122 (PMC8452202; doi:10.36660/abc.20201122)
Supplement: Translation

# Translation: Position Statement on Indications for Echocardiography in Fetal and Pediatric Cardiology and Congenital Heart Disease of the Adult – 2020

**Authors:** Samira Saady Morhy, Silvio Henrique Barberato, Alessandro Cavalcanti Lianza, Andressa Mussi Soares, Gabriela Nunes Leal, Ivan Romero Rivera, Marcia Ferreira Alves Barberato, Vitor Guerra, Zilma Verçosa de Sá Ribeiro, Ricardo Pignatelli, Carlos Eduardo Rochitte, Marcelo Luiz Campos Vieira

**Affiliations:** 1 São PauloSP Brazil Hospital Israelita Albert Einstein, São Paulo, SP – Brazil; 2 CuritibaPR Brazil Cardioeco – Centro de Diagnóstico Cardiovascular, Curitiba, PR – Brazil; 3 CuritibaPR Brazil Quanta Diagnóstico e Terapia, Curitiba, PR – Brazil; 4 São PauloSP Brazil Instituto da Criança e do Adolescente do Hospital das Clínicas da Faculdade de Medicina da Universidade de São Paulo (HC-FMUSP), São Paulo, SP – Brazil; 5 São PauloSP Brazil Hospital do Coração, São Paulo, SP – Brazil; 6 Cachoeiro de ItapemirimES Brazil Hospital Evangélico de Cachoeiro de Itapemirim e Clínica CORImagem, Cachoeiro de Itapemirim, ES – Brazil; 7 São PauloSP Brazil Hospital e Maternidade São Luiz Itaim, São Paulo, SP – Brazil; 8 MaceióAL Brazil Santa Casa de Misericórdia de Maceió, AL – Brazil; 9 Toronto Canada The Hospital for Sick Children, Toronto – Canada; 10 SalvadorBA Brazil Hospital Português, Salvador, BA – Brazil; 11 SalvadorBA Brazil Hospital Aliança, Salvador, BA – Brazil; 12 HoustonTexas USA Texas Children's Hospital, Baylor College of Medicine, Houston, Texas – USA; 13 São PauloSP Brazil Instituto do Coração da Faculdade de Medicina da Universidade de São Paulo (InCor, FMUSP), São Paulo, SP – Brazil

**Development:** Cardiovascular Imaging Department (Departamento de Imagem Cardiovascular – DIC) of the Brazilian Society of Cardiology (Sociedade Brasileira de Cardiologia – SBC) and the Cardiovascular Imaging Society of the Interamerican Society of Cardiology (Sociedad de Imágenes Cardiovasculares de Sociedad Interamericana de Cardiología – Sisiac, Siac)

**Norms and Guidelines Council:** Brivaldo Markman Filho, Antonio Carlos Sobral Sousa, Aurora Felice Castro Issa, Bruno Ramos Nascimento, Harry Correa Filho, Marcelo Luiz Campos Vieira

**Norms and Guidelines Coordinator:** Brivaldo Markman Filho

**Coordinating Editor:** Samira Saady Morhy

**Co-editors:** Silvio Henrique Barberato, Carlos Eduardo Rochitte, Marcelo Luiz Campos Vieira

**Table:** 

Declaration of potential conflict of interests of authors/collaborators of the Position Statement on Indications for Echocardiography in Fetal and Pediatric Cardiology and Congenital Heart Disease of the Adult – 2020							
If, within the last 3 years, the author/collaborator of the statement:							
Names of statement collaborators	Participated in clinical and/or experimental studies sponsored by pharmaceutical or equipment companies related to this statement	Spoke at events or activities sponsored by industry related to this statement	Was (is) a member of a board of advisors or a board of directors of a pharmaceutical or equipment industry	Participated in normative committees of scientific research sponsored by industry	Received personal or institutional funding from industry	Wrote scientific papers in journals sponsored by industry	Owns stocks in industry
Alessandro Cavalcanti Lianza	No	No	No	No	No	No	No
Andressa Mussi Soares	No	No	No	No	No	No	No
Carlos Eduardo Rochitte	No	No	No	No	No	No	No
Gabriela Nunes Leal	No	No	No	No	No	No	No
Ivan Romero Rivera	No	No	No	No	No	No	No
Marcelo Luiz Campos Vieira	No	No	No	No	No	No	No
Marcia Ferreira Alves Barberato	No	No	No	No	No	No	No
Ricardo Pignatelli	No	No	No	No	No	No	No
Samira Saady Morhy	No	No	No	No	No	No	No
Silvio Henrique Barberato	No	No	No	No	No	No	No
Vitor C. Guerra	No	No	No	No	No	No	No
Zilma Verçosa de Sá Ribeiro	No	No	No	No	No	No	No

## 1. Introduction

In accordance with the “Standards for Production of Guidelines, Position Statements, and Standardizations” sanctioned by the Brazilian Society of Cardiology, this document was written to update indications for echocardiography in fetal and pediatric cardiology and congenital heart disease of the adult, and to supplement the recently-published position paper on indications for echocardiography in adults.^1^ The position statement is not intended to be an in-depth review of echocardiography in congenital heart disease, but an indispensable basic guide to support rational clinical decision-making by physicians when ordering examinations. While it takes into consideration the significant technological advances achieved recently in echocardiography, its purpose is not to describe echocardiography methods in detail, but to clearly and concisely summarize the most important situations in which echocardiography is of benefit for diagnosis and/or treatment planning in these groups of patients. In this document, recommendation classes will be presented in accordance with the following definitions:

– Class I: conditions for which there is conclusive evidence or, in the absence thereof, general agreement that the examination procedure is useful and safe.
– Class II: conditions for which there is conflicting evidence and/or divergence of opinion on the utility and/or safety of the examination.
– Class IIa: evidence or opinions favorable to the examination. Most experts approve.
– Class IIb: utility and/or safety less well established, with divergent opinions.
– Class III: conditions for which there is evidence or consensus that the examination is not useful and, in some cases, may even be harmful.

Evidence levels are also presented, defined as follows:

– A: agreement between multiple randomized clinical trials or robust meta-analyses;
– B: less robust meta-analysis data or single randomized clinical study or observational studies;
– C: expert opinion.

All of the tables summarizing recommendations for use of echocardiography in different clinical scenarios will therefore include columns showing recommendation classes and evidence levels

## 2. Fetal Echocardiography

The incidence of congenital heart disease is estimated at 6-12/1,000 live births;^2^^,^^3^ however, it is estimated that fetal prevalence is higher. There are several factors associated with increased risk of congenital heart disease in the fetus, including familial factors and maternal and fetal conditions. Fetal echocardiography is the most important tool for diagnosis of these cardiac pathologies, from the end of the first trimester up to term. The best timing for conducting fetal echocardiography is determined by multiple factors, including the reason for using it and the gestational age at which a cardiac and/or extracardiac abnormality is detected. Echocardiography for screening high-risk pregnancies can be conducted at 18 to 22 weeks' gestation. Considering that initial screening may not detect developing lesions^4^ or arrhythmia,^5^^,^^6^ abnormal findings at routine obstetric consultations should be promptly referred for additional fetal echocardiography examinations.

Fetal echocardiography can be performed at younger gestational ages, including at the end of the first and start of the second trimesters, generally in pregnancies at high risk of congenital heart disease, particularly when elevated nuchal translucency is present on morphological ultrasound in the first trimester.^7^^,^^8^ In the majority of gestations, transabdominal fetal echocardiography provides images of adequate resolution to detect anomalies at between 13 and 14 weeks. However, if the examination is conducted before 13 weeks, transvaginal echocardiography is needed, because of the small size of the cardiac structures and the distance between the fetus and the maternal abdominal wall.^7^^,^^8^ When fetal echocardiography is conducted before 18 weeks, it should be repeated between 18 and 22 weeks' gestation, because the limited image resolution may not be sufficient for diagnosis of certain cardiac abnormalities and also because of potential progression of lesions not detected at earlier gestational ages.^7^^–^^9^

The timing and frequency of echocardiography should be guided by: severity of lesions, signs of heart failure, mechanisms of progression, and perinatal management assessment.

Fetal echocardiography recommendations are listed in Tables 1 and 2.

**Table 1:** Recommendations for fetal echocardiography in high-risk pregnancies^5^^–^^9^

Recommendations	Recommendation class	Evidence level
Pre-gestational DM	I	A
GDM diagnosed in first trimester	II	B
Maternal phenylketonuria	I	A
Maternal SSA/SSB antibodies	IIa	B
Maternal medications: ACE inhibitors Retinoic acid NSAID in third trimester	IIa I I	B B A
Maternal rubella infection in first trimester	I	C
Maternal infection with suspicion of myocarditis/pericarditis	I	C
Assisted reproduction	IIa	A
Congenital heart disease in first-degree relative	I	B
Heart disease with Mendelian inheritance in first or second-degree relative	I	C
Suspicion of heart disease on obstetric ultrasound	I	B
Extracardiac fetal anomaly	I	B
Fetus with chromosome abnormality	I	C
Fetus with tachycardia or bradycardia or frequent irregular heartbeats	I	C
NT > 95%	I	A
Monochorionic twinning	I	A
Fetus with hydrops or effusions	I	B

*ACE: angiotensin-converting enzyme; DM: diabetes mellitus; GDM: gestational diabetes mellitus; NSAID: nonsteroidal anti-inflammatory drugs; NT: nuchal translucency. Adapted from Donafrio et al.*^7^

**Table 2:** Recommendations for fetal echocardiography in low-risk pregnancies^5^^–^^9^

Recommendations	Recommendation class	Evidence level
Maternal medication: Anticonvulsant Lithium Vitamin A Selective serotonin reuptake inhibitors NSAID during first and second trimesters	IIb	B
Heart diseases in second-degree relatives	IIb	B
Abnormalities of the umbilical cord and placenta	IIb	C
Fetal intra-abdominal venous abnormality	IIb	C

*NSAID: nonsteroidal anti-inflammatories. Adapted from Donafrio et al.*^7^

## 3. Echocardiography in the Newborn

Newborn infants transition from a state in which circulation is in parallel, with low systemic vascular resistance and high pulmonary vascular resistance, during fetal life, to a state in which circulation is in series and the cardiac output of both ventricles must be equal in the presence of high systemic vascular resistance. These circulatory changes that take place with birth may take days or weeks to be completed, particularly in preterms, because the communications present during fetal life cannot close promptly. Thus, persistent ductus arteriosus (PDA), persistent high pulmonary pressures, and the incapacity of the immature myocardium to pump blood against systemic vascular resistance that has suddenly increased can cause a transitory reduction in systemic blood flow, changing these patients' hemodynamics.^9^ Moreover, structural cardiac anomalies or extracardiac conditions such as sepsis or diaphragmatic hernia are tolerated differently in this age group.^10^

The transitional physiology of the cardiovascular circulation during the neonatal period means that these patients must be evaluated as a distinct group.

The most common reasons for conducting an echocardiogram during the neonatal period are to detect or rule out congenital structural cardiac diseases in patients who have heart murmur, abnormal neonatal oximetry screening results,^11^ are in shock, are hypoxemic, develop respiratory failure, or have multiple malformations. The next most common group of indications are to screen for functional anomalies, such as persistent ductus arteriosus, and to test pulmonary hemodynamics and cardiac function (see Table 2).

Echocardiographic assessment of patients in neonatal intensive care units is justified, including in an evolving manner, as a factor in specific changes to clinical management of the neonate.

The recommendations for echocardiography in newborn infants are listed in Table 3.

**Table 3:** Recommendations for echocardiography in newborn infants^9^^,^^11^^–^^15^

Recommendations	Recommendation class	Evidence level
Pathological heart murmur or other abnormal cardiac auscultation findings	I	C
Central cyanosis, heart failure, cardiogenic shock, respiratory distress	I	A
Asymmetry of pulses and/or arterial blood pressure gradient between upper and lower extremities	I	A
Cardiomegaly on radiological chest examination or abnormal findings suggestive of heart disease	I	A
Syndromes associated with cardiovascular disease	I	B
Extracardiac anomalies	I	B
Anomaly of heart position or site	I	B
Fetal and/or obstetric echocardiography findings showing or suggesting heart malformation	I	C
Corrective or palliative heart surgery	I	B
History of hydrops fetalis	I	B
Clinical suspicion of patent ductus arteriosus	I	A
Evaluation of the hemodynamic significance of PDA, monitoring effects of treatment	I	A
Assessment of progress of neonate after surgery for closure of ductus arteriosus with hemodynamic instability	I	A
Perinatal asphyxia with abnormal hemodynamics and/or biomarkers	I	A
Suspected pulmonary hypertension	I	A
Assessment of progress of neonate with pulmonary hypertension on drug treatment	I	A
Hypotension	I	A
Assessment of extracorporeal life support cannulae, maintenance and weaning from ECMO	I	A
Systemic maternal disease associated with known neonatal anomaly	IIa	B
Maternal infection during gestation or delivery with potential for fetal or neonatal cardiac sequelae	IIa	B
Maternal diabetes without fetal echocardiography or with normal fetal echocardiography	IIb	B
Maternal phenylketonuria	I	A
Maternal autoimmune dysfunction	IIa	B
Maternal exposure to teratogens	IIa	B
Failure to thrive in the absence of definite clinical abnormalities	IIa	C
History of nonsustained fetal ectopic heart rhythm, in the absence of postpartum arrhythmia	III	C
Acrocyanosis with normal pulse oximeter saturation in upper and lower extremities	III	C
Morphological and functional assessment during the postoperative period after heart surgery	I	B
To assess pericardial hemorrhage and evaluate hemodynamic impact and guide interventional procedures	I	A
To determine central venous catheter position and identify related complications (thrombosis and infection)	I	A

*ECMO: extracorporeal membrane oxygenation; PDA: persistent ductus arteriosus.*

## 4. Echocardiography in Infants, Children and Adolescents

Since echocardiography is a noninvasive method for obtaining anatomic, hemodynamic, and physiological information on the pediatric heart, it is the first-choice diagnostic method for initial assessment of congenital or acquired heart disease in infants, children, and adolescents.

Children with cardiac diseases are a varied group of patients who often have complex anatomic malformations and require lifelong follow-up. Repeated studies may therefore be indicated to monitor heart valve function, growth of cardiovascular structures, and ventricular function and for follow-up of drug-based or surgical interventions.^9^^,^^16^^–^^18^

Signs and symptoms such as cyanosis, growth deficits, exercise-induced anginas, syncope, respiratory distress, murmurs, heart failure, pulse abnormalities, and cardiomegaly may suggest structural heart disease.

Echocardiography may also be indicated even in the absence of specific clinical status in patients with family history of hereditary heart disease, genetic syndromes associated with structural heart disease, or abnormal examination findings (fetal echocardiography, chest X-ray, and electrocardiogram).

Patients with arrhythmia may have structural heart disease, such as corrected transposition of the great arteries and Ebstein's anomaly, cardiac tumors, or cardiomyopathies. Sustained arrhythmia and use of antiarrhythmic medications can cause changes to myocardial function and echocardiography plays an important role in clinical management of these patients.

The recommendations for echocardiography in infants, children and adolescents are listed in Table 4.

**Table 4:** Recommendations for echocardiography in infants, children and adolescents^9^^,^^12^^,^^16^^–^^18^

Recommendations	Recommendation class	Evidence level
Pathological heart murmur or other evidence of cardiac abnormality	I	C
Anomaly of heart position or site	I	B
Cardiomegaly on radiological chest examination or abnormal findings suggestive of heart disease	I	B
Abnormal electrocardiogram	I	B
Immediate preoperative assessment for heart surgery	I	C
Change in clinical status of patient with known heart disease	I	B
Morphological and functional assessment during the postoperative period after heart surgery	I	C
Family history of heart disease transmitted genetically	I	B
Neuromuscular disease with myocardial involvement	I	B
Signs and symptoms of infectious endocarditis	I	A
Signs and symptoms of heart failure	I	A
Palpitations without other symptoms, benign family history, and normal electrocardiogram	IIb	C
Palpitations with family history of arrhythmia, sudden death, or cardiomyopathy.	I	B
Palpitations in patient with known cardiomyopathy	I	B
Palpitations with abnormal electrocardiogram or known ion channel defects	IIa	C
Asymmetry of peripheral pulses	I	A
Syndrome associated with cardiovascular disease; genotype positive for cardiomyopathy; chromosome anomaly associated with cardiovascular disease	I	B
To determine the appropriate timing of clinical or surgical treatment in patients with known heart disease	I	B
Selection, placement, patency, and monitoring of endovascular devices	I	A
Identification of intracardiac and intravascular shunts before, during, and after interventional percutaneous cardiac catheterization	I	A
Prolonged fever, without apparent cause, in a patient with congenital heart disease	I	A
Functional murmur in an asymptomatic patient	IIb	C
Retarded growth in the absence of specific clinical abnormality	IIb	C
Atypical angina, identified as of musculoskeletal origin in an asymptomatic patient	III	
Syncope with abnormal electrocardiogram, exercise-related syncope	I	A
Syncope with family history of cardiomyopathy or sudden death	I	A
Neurocardiogenic (vasovagal) syncope	IIa	C
Effort angina or angina at rest with abnormal electrocardiogram	I	B
Angina associated with fever or use of illicit drugs	IIa	B
Presumably innocent murmur with signs and symptoms of heart disease	I	C
Central cyanosis	I	A
Chest wall deformity and preoperative scoliosis	IIb	C
Extracorporeal life support: initiation, maintenance, and weaning	I	B
Previous normal echocardiography with change in cardiovascular status and/or new family history suggestive of hereditary heart disease	IIa	C
Abnormal cardiac biomarkers	I	B
Hemoglobinopathies	I	B
Connective tissue diseases (Marfan, Loeys, Dietz, and others)	I	B
Muscular dystrophy	I	B
Autoimmune diseases	I	B
Arterial hypertension	I	A
Stroke	I	B
Metabolic, mitochondrial, or storage disease	I	B
Family history of cardiovascular disease: sudden death before 50 years of age, connective tissue diseases (Marfan or Loeys Dietz syndromes), idiopathic arterial hypertension	IIa	C
Family history of cardiovascular disease: hypertrophic cardiomyopathy, nonischemic dilated cardiomyopathy, hereditary pulmonary arterial hypertension	IIa	B

## 5. Pediatric Echocardiography in Acquired Heart Diseases

Acquired heart diseases primarily occur in the context of systemic diseases linked to inflammatory processes, renal diseases, use of cardiotoxic chemotherapy, or parenchymatous pulmonary disease, and after heart transplantation.

Myocardial involvement can occur in several conditions, such as systemic inflammatory diseases (particularly those with a more aggressive course, such as juvenile systemic lupus erythematosus, juvenile idiopathic arthritis, and rheumatic fever).^19^^–^^22^ During treatment with cardiotoxic chemotherapy (particularly with anthracyclines) and radiotherapy in the mediastinal region, echocardiography is indicated before, during, and after treatment, with the objective of indicating the need for cardioprotective measures and even for changing the treatment in some cases.^23^

In patients with chronic liver disease or hypertension and/or on dialysis, echocardiography provides clinicians with valuable information on ventricular geometry, systolic/diastolic function, and blood volume. This can very often guide changes in the dialysis regimen and introduction of (or changes to) antihypertensive and vasoactive drugs.^24^

In patients with pulmonary disease, echocardiography can be used to estimate pulmonary pressures and also to evaluate right ventricle performance, which has an important correlation with clinical prognosis.^25^^–^^27^

In children and adolescents with AIDS, echocardiography is used to investigate right cardiac involvement caused by the virus, which can result in dilated cardiomyopathy, pulmonary hypertension, and even ventricular hypertrophy, in addition to effects caused by opportunistic diseases and/or drug side effects.^28^

The growing number of children with end-stage heart failure must be evaluated before and after heart and/or cardiopulmonary transplantation^29^ and echocardiography is also an aid to decision-making on introduction/withdrawal of cardiovascular support.^30^

The recommendations for echocardiography in newborn infants, infants, children, and adolescents with acquired heart disease are listed in Table 5.

**Table 5:** Recommendations for echocardiography in newborn infants, infants, children, and adolescents with acquired heart disease^9^^,^^16^^–^^31^

Recommendations	Recommendation class	Evidence level
Initial assessment and reassessments in patients with suspected or confirmed diagnosis of Kawasaki syndrome, Takayasu's Arteritis, myopericarditis, AIDS, and rheumatic fever	I	B
After heart or cardiopulmonary transplantation	I	B
Initial assessment and reassessments in patients treated with cardiotoxic chemotherapy and mediastinal radiotherapy	I	B
Initial assessment and reassessments in patients with myocardial disease	I	C
Assessment of cardiac involvement in severe kidney disease and/or systemic arterial hypertension	I	B
Assessment of donors for heart transplantation	I	C
Pulmonary arterial hypertension	I	A
Assessment of progression of pulmonary arterial hypertension treated with drugs or surgery	I	B
Initiation or withdrawal of extracorporeal cardiopulmonary support	I	C
Thromboembolic event	I	C
Sepsis, right heart failure, or cyanosis in a patient with venous catheter	I	B
Systemic or pulmonary embolization in a patient with right-left flow and venous catheter	I	C
Superior vena cava syndrome in a patient with venous catheter	I	C
Liver disease	IIa	C
Obesity with other cardiovascular risk factors or obstructive sleep apnea	IIa	C
Sepsis	IIa	B
Cystic fibrosis without evidence of cor pulmonale	IIa	C
Follow-up of patients after rheumatic fever without evidence of cardiac involvement	IIb	C
Cardiac assessment after pericarditis without evidence of recurrent pericarditis or chronic pericarditis	IIb	C
Fever in a patient with venous catheter without evidence of systemic or pulmonary embolization	IIb	C
Routine assessment for participation in competitive sports in patients with normal cardiovascular examination	IIb	C
Late follow-up of Kawasaki syndrome without evidence of coronary abnormalities in the acute phase	III	C
Routine assessment in an asymptomatic patient with venous catheter	III	C

## 6. Echocardiography in Adults with Congenital Heart Disease

Over the last 30 years, considerable advances were made in pediatric cardiology, both in the sphere of diagnosis with the advent of echocardiography and in the realm of treatment to correct heart diseases, initially surgically and more recently using percutaneous techniques in the catheterization laboratory. Recent data show that the estimated size of the population of adults with congenital heart disease in United States in 2010 was 1.4 million patients.^30^ This population has problems related to residual defects, new acquired defects (such as pulmonary reflux after definitive correction of tetralogy of Fallot or obstructions after a Jatene procedure), arrhythmia, heart failure, acquired disease of the adult, infectious endocarditis, or indications for heart transplantation. Many survive with palliative surgery that may or may not require definitive correction (such as the Senning, Mustard, Rastelli, Glenn, or Fontan procedures, which induce new complications that are implicit in the surgical method employed) and many patients present with heart conditions for the first time, with no prior diagnosis of heart disease.^32^^–^^35^

There is no doubt that two-dimensional transthoracic echocardiography has an important role to play in diagnosis and follow-up of these malformations.^36^ Recent advances such as 3D echocardiography have proved superior for determination of volumes and even ventricular function, particularly in complex malformations such as those with univentricular physiology, or for evaluation of the right ventricle, and these systems should be used whenever they are available and there are trained professionals to operated them.^37^ Additionally, using 3D images to guide surgery gives surgeons better understanding of the case, enabling better surgical planning. Along the same lines, new techniques for assessment of diastolic function and segmental function, such as tissue Doppler, strain, and strain rate can be very useful, particularly in conditions with univentricular physiology or cardiac chamber deformities, primarily when involving the right ventricle^38^ (see sections 9 and 10 below).

The primary limitation of echocardiography for assessment of adults with congenital heart disease is a poor transthoracic acoustic window in patients with previous heart surgery or deformities of the chest wall, and echocardiography is also inappropriate for assessing the aortic arch, the coronary arteries, the pulmonary arteries, and the collateral vessels. In these situations, transesophageal echocardiography, angiotomography, and magnetic resonance (MR) are extremely useful.

The recommendations for echocardiography in adults with congenital heart disease are listed in Table 6.

**Table 6:** Recommendations for echocardiography in adults with congenital heart disease^9^^,^^29^^,^^36^^,^^38^^–^^44^

Recommendations	Recommendation class	Evidence level
Initial structural and functional assessment in suspected congenital heart disease because of murmur, cyanosis, poor arterial saturation, or abnormal electrocardiogram or chest X-ray findings	I	C
Changes in the clinical status of a patient with known congenital heart disease, whether operated or not	I	C
Doubts with regard to original diagnosis or unexplained structural or hemodynamic abnormalities in a patient with known congenital heart disease	I	C
Follow-up of patients with intraventricular communication for assessment of evolving morphological changes	I	C
Periodic follow-up of patients with congenital heart disease, operated or not, in whom assessment of contraction, valve, and conduction function is needed	I	C
Postoperative annual follow-up after total, partial, or palliative repair in patients with residual defects and sequelae that could compromise clinical progress	I	C
Identification of the origin and initial course of the coronary arteries	I	C
Assessment of unexplained post-exercise syncope for initial diagnostic definition	I	C
Evaluation of aortic injury in patients with suspected or confirmed Marfan Syndrome for serial assessment of the aorta and/or mitral valve	I	C
Periodic examinations in patients operated for PDA, ASD, VSD aortic coarction or bicuspid aortic valve, without residual defect and without changes in clinical condition	III	C
Follow-up of patients with heart diseases without hemodynamic significance and without changes in clinical condition	III	C
Assessment of lesions in the aortic arch, pulmonary arteries and collateral arteries, the anatomy of which is better defined using other diagnostic methods	III	C
Periodic assessment of cardiac malformations without changes in physical examination findings, in the clinical condition of the patient, or in other examinations such as electrocardiogram and chest X-ray	III	C

*ASD: atrial septal defect; PDA: patent ductus arteriosus; VSD: ventricular septal defect.*

## 7. Transesophageal Echocardiography in Pediatric Cardiology

Transesophageal echocardiography (TEE) uses special transducers and a different access route, offering better definition of cardiac structures, increasing the method's diagnostic applications.

It is particularly important for definition of complex anatomic structures and functional abnormalities, which cannot always be evaluated using transthoracic echocardiography alone.

Technological advances and miniaturization of probes has led to increasing adoption of TEE in the field of pediatric cardiology and it can provide important information about patients from the neonatal age group up to adolescents and adults, for diagnosis, intraoperative assessment, in the immediate and late postoperative periods, and in the intensive care unit, and also in the catheterization laboratory, aiding in interventional procedures.

### 7.1. Transesophageal Echocardiography as a Diagnostic Tool

Transesophageal echocardiography should be adopted to improve diagnostic definition of heart disease in situations in which better anatomic evaluation is needed in certain specific congenital heart diseases, in the majority of cases in adults, since in children the image quality of transthoracic echocardiography is generally good (Table 7).

**Table 7:** Recommendations for transesophageal echocardiography as a diagnostic tool^9^^,^^45^

Recommendations	Recommendation class	Evidence level
Confirmation or exclusion of a relevant clinical diagnostic suspicion not observable using TTE	I	A
Insufficient anatomic and hemodynamic information using TEE, primarily in children with chest deformities or obesity and in adults with congenital heart disease	I	A
Assessment of PFO as a possible etiology of central or peripheral embolic events in young patients (< 60 years), with agitated saline contrast to determine the possibility of right-left flow. To assess PFO risk factors for stroke/TIA: interatrial septum aneurysm, passage of > 30 microbubbles from right atrium to left atrium, PFO tunnel > 10 mm, and prominent Eustachian valve	I	A
Assessment of PFO before placement of a transvenous pacemaker	I	A
Classification, dimensions, and location of atrial septal defect, primarily in adult patients and those with poor transthoracic definition for selection of possible candidates for percutaneous occlusion and choice of occlusion device.	I	A
Assessment of aortic dissection in Marfan, Ehlers-Danlos, and Turner syndromes and in aortic coarctation	I	A
Assessment of the aorta in the Takayasu's Arteritis	I	A
Assessment of the intra or extra-cardiac tubes during the postoperative period after Senning, Mustard, or Fontan procedures	I	A
Assessment of thrombi, masses, vegetations, abscesses, and prostheses	I	A
For determination of the degree and mechanisms of mitral valve reflux to aid in surgical or percutaneous repair (Mitraclip)	I	B

*PFO: patent foramen ovale; TEE: transesophageal echocardiogram; TIA: transient ischemic attack; TTE: transthoracic echocardiogram.*

### 7.2. Intraoperative Transesophageal Echocardiography

The most important impact of transesophageal echocardiography in the operating room is detection of significant residual defects that are very often unsuspected. Several authors have reported putting patients back on extracorporeal circulation to review surgery after intraoperative TEE, with rates that vary from 6 to 11.4% of cases, in the different series analyzed.^46^

The indications for intraoperative TEE for congenital heart disease are listed in Table 8.

**Table 8:** Recommendations for intraoperative transesophageal echocardiography^9^^,^^45^^–^^46^

Recommendations	Recommendation class	Evidence level
Perioperative assessment of cardiac anatomy and function	I	A
Monitoring of surgical procedures involving risk of abnormal flows, valve reflux, residual obstructions, or myocardial ventricular dysfunction	I	A
Minimally invasive surgery, video-guided surgery, and hybrid procedures	I	A

### 7.3. Transesophageal Echocardiography in the Intensive Care Unit (ICU)

In the immediate postoperative period, the definition of TEE images may be compromised by drains, dressings, meshes, and mechanical ventilation, making it necessary to use TEE, which can provide anatomic (residual lesions) and hemodynamic information that is important for clinical and therapeutic management of patients (Table 9).

**Table 9:** Recommendations for transesophageal echocardiography in the ICU^9^^,^^45^

Recommendation	Recommendation class	Evidence level
Assessment of residual defects, pericardial hemorrhage, and ventricular function in patients with a poor transthoracic acoustic window	I	A
Postoperative monitoring in a patient with an open sternum	I	A

### 7.4. Transesophageal Echocardiography in the Catheterization Laboratory

Transesophageal echocardiography is helpful during hemodynamic interventions, providing diagnostic details in a range of heart diseases and for monitoring procedures, in addition to providing anatomic information on the results and on possible residual lesions^47^ (Table 10).

**Table 10:** Recommendations for Transesophageal Echocardiography in the Catheterization Laboratory^9^^,^^45^^,^^47^

Recommendation	Recommendation class	Evidence level
In percutaneous closure of patent foramen ovale, interatrial and interventricular communications	I	A
Postoperative closure of fenestrations after Fontan procedures	I	A
During dilatation of Senning and Mustard procedure tunneling	I	A
During stenting of stenosis of pulmonary arteries and tubes	IIb	B
For guidance in mitral valvoplasty and percutaneous mitral valve repair (Mitraclip)	I	A
For guidance in pulmonary and aortic valvoplasties	IIa	A
Placement of aortic endoprostheses to treat aneurysms, dissections, hematoma, or parietal ulcers of the thoracic aorta	I	A
Catheter guidance for perforation and percutaneous dilatation of atretic valves	I	A
During therapeutic interventional catheterization for radio frequency ablation	I	A

## 8. Stress Echocardiography in Pediatric Cardiology

Echocardiography under stress (physical or pharmacological) is a well-established technique in adults.^48^^,^^49^ There are not yet specific guidelines or recommendations for the pediatric age group. However, as in the adult population, applications in children and adolescents have been concentrated on investigation of ischemic disease,^50^^–^^56^ but are being extended to other areas that are not necessarily ischemic^55^^–^^63^ (Table 7).

Both types of stress, pharmacological and exercise, can be administered to children, with certain peculiarities.^64^^–^^66^ Dobutamine is the most common pharmacological agent and is used in the same protocols as with adult patients. In general, sedation or even anesthesia is recommended for children under the age of 8. Physical exercise can be used with children over the age of 8 who are cooperative and able to exercise on a treadmill or bicycle.^67^

## 9. Three-dimensional Echocardiography

Three-dimensional (3D) echocardiography has been incorporated into clinical practice, providing additional information in comparison to two-dimensional (2D) echocardiography, and is primarily used for congenital defects in which the three-dimensional view offers images very close to the anatomic and surgical planes.^68^ The same concept is applicable to procedures undertaken in the catheterization laboratory, in which the three-dimensional view can be used not only to guide the procedures, but also to better evaluate the anatomy when choosing which devices to employ. Assessment of ventricular volumes and function has also been performed using the 3D technology, primarily to evaluate ventricular geometry in the most diverse forms of congenital defects, including univentricular hearts.^69^^,^^70^ Atrioventricular valves can be assessed not only from the point of view of anatomic details, including the subvalvular apparatus, but also in terms of functional assessment of valve ring movement, and interactions between movement of valve leaflets and chords.^71^

When dealing with pediatric patients, the larger transthoracic acoustic window is a great advantage. More recently, more advanced transducers have been developed with a smaller footprint and higher frequency (2 to 8 MHz). However, the image quality is still not the same when a 2D-3D combination is used with the same transducer, particularly in small patients. Another significant challenge that remains to be overcome is development of a pediatric transesophageal transducer, which limits 3D TEE to use in patients weighing more than 30 kg, according to manufacturers' recommendations. In small children, use of a pediatric transducer with higher frequency is recommended, as well as the epicardial echocardiogram, for intraoperative scenarios. Three-dimensional transesophageal echocardiography should always by considered in larger patients (generally weighing more than 30 kg) if transthoracic 3D imaging does not yield sufficient information to plan surgery or other interventions.

In a variety of different congenital defects, 3D echocardiography can provide additional information on a wide range of anatomic structures, including atrial and ventricular septa, the semilunar and atrioventricular valves, and also the outflow tracts. Applications are expanding as technological progress advances and adaptations are made to suit the pediatric population. Currently, use is based more on clinical need for additional information than on randomized studies showing the advantage of 3D over 2D. Use is therefore individualized and depends on the profile of the imaging laboratory or hospital adopting the technology for specific lesions.

Valve lesions and isolated septal defects are the principal indications. However, in situations in which there are concomitant anomalies of the ventriculoarterial connection, as in double-outlet right ventricle, the position and size of the intraventricular communication can be better visualized and demonstrated with 3D echocardiography.

Depending on the area or structure assessed by transthoracic and/or transesophageal 3D echocardiography, it may provide relevant information that complements the findings of 2D echocardiography.^72^^–^^83^ Little additional information is yielded by using 3D echocardiography to assess the pulmonary arteries, the pulmonary valve, and even the right ventricle outflow tract and the aortic arch (Table 12).

**Table 11:** Recommendations for stress echocardiography in pediatric cardiology

Recommendation	Recommendation class	Evidence level
To investigate coronary failure in children after late heart transplantation	IIa	B
Late assessment in Kawasaki disease with coronary abnormalities in the acute phase	IIa	B
During the postoperative period after Jatene procedure and the postoperative periods of abnormal origin and course of coronary arteries, and coronary-cameral fistulae	IIa	B
Ventricular function in myocardiopathy and mitral and aortic valve failure	IIa	B
Screening for ventricular dysfunction in patients treated with chemotherapy regimens including anthracyclines and after transplant, to test myocardial function during exercise	IIa	B
To investigate coronary failure in children with pulmonary atresia with intact ventricular septum, dyslipidemia, insulin-dependent diabetes mellitus, or supravalvular aortic stenosis	IIb	B
Evaluation of pressure gradient behavior in hypertrophic cardiomyopathy and pulmonary and aortic valve stenosis	IIb	B
Evaluation of myocardial reserve in the late postoperative period after atrial switch surgery for great vessel transposition, right ventricle assessment in late postoperative period of tetralogy of Fallot surgery	IIb	B

**Table 12:** Additional information yielded by 3D echocardiography on specific anatomic structures and recommendations^72^^–^^78^^,^^80^^–^^82^^,^^87^^,^^88^^,^^91^

Anatomic structure of interest	Modality	Additional information	Recommendation class	Evidence level
Interatrial septum	TTE/TEE	Dimension, format, and location of defect(s)	I Complex or residual defects II Central and single defects	B B
Tricuspid valve	TTE/TEE	Morphology of leaflets, subvalvular apparatus (chords), location of regurgitation jets	I	B
Mitral valve	TTE/TEE	Morphology of leaflets, subvalvular apparatus (chords), location of regurgitation jets	I	B
Interventricular septum	TTE/TEE	Dimension, format, and location of complex defect(s)	I	B
LV outflow tract	TTE/TEE	Morphology of subaortic obstruction	I	B
Aortic valve	TTE/TEE	Aortic valve measurements, morphology of leaflets, regurgitation mechanism	I	B
RV outflow tract	TTE/TEE	Morphology and visualization of site of obstruction	III	C
Pulmonary valve	TTE	Morphology	IIa	C

*LV: left ventricle; RV: right ventricle; TEE: transesophageal echocardiogram; TTE: transthoracic echocardiogram.*

Three-dimensional echocardiography can provide additional information in the context of certain specific congenital heart disease in which there are connection anomalies (atrioventricular or ventriculoarterial)^76^^,^^84^^–^^86^ (Table 13).

**Table 13:** Additional information yielded by 3D echocardiography on congenital defects and recommendations71,79,83-86

Congenital heart disease	Modality	Additional information	Recommendation class	Evidence level
AVSD	TTE/TEE	Dimension of atrial and/or ventricular defect; morphology of leaflets and subvalvular apparatus; assessment of regurgitation jets; dimensions of orifices and ventricles in unbalanced defects	I	B
Discordant AV connection	TTE/TEE	Morphology and function of tricuspid and mitral valves, location and dimensions of related VSD morphology of outflow tracts of the RV and LV	I	B
Complex TGA	TTE/TEE	Morphology and function of tricuspid and mitral valves, location and dimensions of the VSD, anatomy of RV and LV outflow tracts in cases of obstruction	I	B
Tetralogy of Fallot	TTE	Dimension and location of CIV and anatomy of RV outflow tract	III	C
Truncus Arteriosus	TTE/TEE	Morphology of truncal valve*	III	C
Double-outlet RV	TTE	Relationship of atrioventricular valves, position and size of the VSD with the great arteries	III	C

*AV: atrioventricular; AVSD: atrioventricular septal defect; TEE: transesophageal echocardiogram; TGA: transposition of the great arteries; TTE: transthoracic echocardiogram; VSD: ventricular septal defect.*

* *Specifically for assessment of the truncal valve in older patients.*

Application of 3D echocardiography in the catheterization laboratory for closure of atrial and ventricular septal defects complements 2D images for delimiting the margins of defects and related structures,^87^^,^^88^ specifically in atrial communications of the type ostium secundum, which are very well demonstrated by real-time imaging with 3D transesophageal echocardiography. Closure of interventricular communications using percutaneous or transmural devices can also be guided and, primarily, assess nearby structures, such as, for example, leaflets and/or tricuspid valve chords. There are other applications in the catheterization laboratory in which 3D echocardiography can be used to guide procedures: closure of fenestrations in the Fontan procedure, coronary fistulae, ruptures of the sinus of Valsalva, paravalvular regurgitation, septal perforation, and location of electrodes for cardiac resynchronization.^89^^–^^94^

A major challenge in congenital heart disease is evaluation of ventricular volumes and function, because of reasons that are intrinsic to the congenital defects involved (position of the heart, connection anomalies, non-contractile material, and differences in ventricular preload, among others). The software packages available were developed on the basis of the left ventricular geometry of normal hearts, which can often invalidate the information obtained using 3D systems. Although measurements of volumes and ejection fractions are replicable, 3D echocardiography has shown smaller volumes than MR when quantifying volumes, which prevents one from being substituted for the other. As a result, clinical application is still complicated by the absense of values for normality in the pediatric population. It is not recommended that software developed for the normal left or right ventricle be used with congenitally malformed ventricles until new software or models have been validated.^70^^,^^95^^–^^97^

The general recommendation for use of 3D transthoracic echocardiography in pediatrics is that the decision should be taken in accordance with the type of patient and the profile of the echocardiography laboratory and/or hospital.

There is consensus that 3D is a modality that complements rather than substitutes 2D echocardiography, irrespective of the type of disorder.

## 10. Myocardial Deformation Imaging in Pediatric Patients

Myocardial deformation (strain) is proving to be a useful tool for evaluation of diastolic and systolic function, in both adults and the pediatric population.^98^ Myocardial strain analysis by speckle tracking imaging is a method that is independent of the angle of insonation and has low intraobserver and interobserver variability, enabling global and regional ventricular function to be quantified more accurately than with more traditional methods, such as tissue Doppler, fractional shortening, or ejection fraction.^99^ Some studies have shown that strain obtained by speckle tracking has high prognostic value, underscoring its utility for both congenital and acquired pathologies.^100^

Notwithstanding, myocardial strain is subject to physiological variations caused by age, sex, heart rate, preload, arterial blood pressure, and body surface area, in addition to the type of software used for the analysis.^101^ Efforts are ongoing to establish normal values for strain that can be used as a universal reference in pediatrics, so that myocardial deformation analysis can be incorporated into guidelines and start to be adopted in clinical routines.^102^^–^^104^ Meanwhile, myocardial deformation imaging has recommendation class II and evidence level B for use in the many different pediatric diseases.

### 10.1. Ventricular Strain in Acquired Heart Diseases in Childhood

Analysis of right and left ventricular strain is particularly useful in situations in which the intention is to identify systolic and/or diastolic dysfunction while in the subclinical phase. The information obtained from strain analysis makes opportune therapeutic intervention possible in a range of systemic diseases with myocardial involvement.

Early detection of myocardial damage secondary to use of anthracyclines is one of the most important contributions of myocardial deformation imaging to date and has been incorporated into protocols for monitoring patients in oncology.^105^^–^^108^

A correlation has been demonstrated between the degree of inflammatory activity and the values of LV strain and systolic and diastolic LV strain rate in patients with rheumatic diseases, such as childhood-onset systemic lupus erythematosus.^20^

Other studies have confirmed the efficacy of strain obtained using the speckle tracking technique for detection of myocarditis of both autoimmune and viral etiology.^109^^,^^110^ In cases of dilated cardiomyopathy in children, the pattern of regional compromise of LV strain influenced the outcome of death or transplantation, as demonstrated by Forsha et al.^111^ Another use for strain in cases of dilated cardiomyopathy is to detect dyssynchrony, identifying cases that could benefit from resynchronization.^111^

After orthotopic heart transplantation in children, strain analysis has reasonable sensitivity and specificity for identifying which individuals will manifest vascular graft disease in later years.^112^ Some reports, including small numbers of transplanted children, suggest there is an association between reduced segmental strain and rejection in endomyocardial biopsies, suggesting the technique could become a less invasive diagnostic instrument in the near future.^113^^–^^115^

In young patients with Duchenne muscular dystrophy, studies have demonstrated a significant reduction in longitudinal and radial strain of the inferolateral and anterolateral walls of the LV, even before ejection fraction is compromised or symptoms of heart failure emerge.^116^ Several studies have demonstrated improved cardiovascular performance and 10-year survival in patients with Duchenne muscular dystrophy who were put on angiotensin-converting enzyme inhibitors and beta blockers as soon as the first echocardiographic signs of myocardial deterioration were detected, while still asymptomatic from a cardiovascular point of view.^117^

Myocardial strain imaging can also contribute to detection of myocardial compromise in storage disorders such as the mucopolysaccharidoses (MPS)^118^ and Pompe disease.^119^ Studies have focused attention on myocardial strain as a parameter for assessment of the impact of long-term enzyme replacement on the ventricular function of patients with these diseases.^120^

Myocardial strain analysis has also emerged as a possible method for early diagnosis of myocardial inflammation and ventricular dysfunction in Kawasaki disease.^51^ McCandless et al.^121^ found evidence that longitudinal LV strain was reduced on initial echocardiograms of patients with Kawasaki who later developed coronary dilation or exhibited resistance to treatment with immunoglobulin. These findings suggest that LV strain could soon come to be used as a tool for risk stratification in Kawasaki patients.^121^

In cases of myocardial dysfunction induced by pediatric sepsis, LV longitudinal and circumferential strain appear to already be reduced in the initial phases, even though ejection fraction is still unimpaired.^122^

In adult patients with chronic renal failure (CRF), reduction of LV longitudinal strain has been confirmed even in initial stages of the disease and with unimpaired ejection fraction. This early compromise of myocardial deformation has been attributed to fibrosis induced by chronic inflammation and uremic toxins. Additionally, the endothelial dysfunction that occurs in CRF may cause an inappropriate vasodilator response, leading to ischemia in an already hypertrophic ventricle. Similar findings have also been documented in pediatric populations, although it remains to be established whether this reduction in longitudinal LV strain can be used as a specific predictor of morbidity and mortality in children with CRF.^123^

Cardiovascular disorders are common among people with HIV infection, but are frequently underdiagnosed and left untreated, which impacts on patients' quality of life and on long-term mortality. They have been attributed both to the direct effects of the virus and to the effects of antiretroviral medications on the myocardium and vasculature. Symptomatic systolic dysfunction is normally only observed in more advanced cases of the acquired immunodeficiency syndrome.^124^ More recent studies with children and young adults confirm compromised longitudinal RV and LV strain, in patients who are still asymptomatic and have normal LV ejection fraction. In 2016, these results prompted Naami et al. to suggest that myocardial deformation imaging should be included in echocardiographic examinations of pediatric patients with HIV, with the objective of identifying patients with subclinical dysfunction and increased cardiovascular risk.^125^

In a study that enrolled adolescents and young adults with thalassemia who underwent multiple transfusions, Chen et al.^126^ identified a negative correlation between serum ferritin and longitudinal LV strain. Additionally, even after correction for sex, age, serum ferritin, and ventricular mass index, longitudinal LV strain remained an independent predictor of adverse events in thalassemic patients, such as heart failure, arrhythmia, and death (HR: 6.05; *p* = 0.033).^127^

Okumura et al. investigated children and adolescents with idiopathic pulmonary hypertension (IPH), confirming the prognostic value of serial assessment of longitudinal RV strain in the pediatric population. A strain value lower than −14% on the initial echocardiogram identified patients who progressed to lung transplant or death with 100% sensitivity and 54.5% specificity. They concluded that myocardial deformation in pediatric IPH is a more sensitive tool than conventional parameters for evaluation of RV function (TAPSE – tricuspid annular plane systolic excursion, FAC – fractional area change, tricuspid S wave velocity) to detect patients with worse prognosis.^127^ In a recent publication, Hooper et al.^128^ confirmed the utility of longitudinal RV strain in clinical follow-up of IPH in children, demonstrating that strain values had an excellent correlation with BNP – B-type natriuretic peptide values, in the course of treatment with prostacyclin analogues.^13^ Table 14 lists recommendation classes and evidence levels.

**Table 14:** Recommendations for ventricular strain analysis in acquired heart diseases of childhood^20^^,^^51^^,^^105^^–^^128^

Indication	Recommendation class	Evidence level
Cardiotoxicity in pediatric oncology	IIa	B
Autoimmune and viral myocarditis	IIa	B
Dilated cardiomyopathy: selection for resynchronization therapy	IIa	B
Vascular graft disease after heart transplantation	IIb	B
Rejection after heart transplantation	IIb	B
Muscular dystrophies (e.g. Duchenne)	IIa	B
Storage diseases (e.g. Pompe and MPS)	IIa	B
Kawasaki disease	IIa	C
Sepsis	IIb	B
Chronic renal failure	IIb	B
HIV/AIDS infection	IIa	B
Chronic anemias (e.g. thalassemia)	IIa	B
Pulmonary hypertension	IIa	B

*AIDS: acquired immunodeficiency syndrome; HIV: human immunodeficiency virus; MPS: mucopolysaccharidoses.*

### 10.2. Ventricular Strain in Congenital Heart Disease

Analysis of longitudinal RV strain in a subpulmonary position proved feasible and reproducible for perioperative assessment of several congenital heart disorders.^129^ However, in the presence of significant residual obstruction during the postoperative period (PO), parameters for evaluation of the longitudinal RV systolic function, such as TAPSE, S wave velocity, and longitudinal peak systolic strain, did not exhibit adequate correlations with ejection fraction according to MR. In situations with residual pulmonary stenosis or a combination of stenosis and pulmonary failure, RV hypertrophy causes a predominance of circumferential fibers, changing the deformation pattern of this chamber, which is habitually more dependent on longitudinal fibers.^130^ Hayabuchi et al.^131^ evaluated RV free wall circumferential peak systolic strain in the subcostal view, specifically in children with congenital heart disease with RV pressure overload. Using this method, they found a better correlation between strain values and ejection fraction in the RV.^131^ Studies with asymptomatic children in the late postoperative period after surgery for tetralogy of Fallot (T4F) identified compromised biventricular longitudinal systolic peak strain. Some authors found a negative correlation between RV longitudinal systolic peak strain and RV ejection fraction and the pulmonary regurgitation fraction, both estimated by MR.^132^ Other studies have documented a negative correlation between LV longitudinal strain and the degree of pulmonary regurgitation, emphasizing the importance of ventricle interdependence.^133^ Although myocardial deformation imaging can detect subclinical systolic dysfunction in postoperative T4F patients who progress to pulmonary regurgitation, unfortunately there is not yet any consensus on a strain cutoff value that can indicate the best timing for pulmonary valve replacement.

Patients with the RV in the systemic position also exhibit abnormal myocardial deformation patterns, with predominance of contraction of circumferential fibers. In this condition, the discrete reduction of longitudinal strain is indicative of changes to right ventricular geometry, and not of true systolic dysfunction. This is an adaptive mechanism, which makes contractility of the systemic RV similar to LV contractility. Recent publications therefore suggest a normal range of longitudinal systolic peak strain values in systemic RV that are below those expected for subpulmonary RV (–10% to –14.5%).^130^ Longitudinal RV strain values below –10% have been associated with occurrence of adverse events, in the late PO after Senning procedures.^134^

Selection of patients with a single ventricle (SV) for Fontan procedure surgery takes into consideration pulmonary vascular resistance and end-diastolic ventricular pressure. However, current indication criteria have proved fallible for a considerable proportion of these patients, who are subject to complications and extended hospital stays. When associated with pulmonary vascular resistance and end-diastolic ventricular pressure, the preoperative circumferential strain rate improves risk stratification for patients with SV who are candidates for Fontan surgery, irrespective of whether the ventricle has right or left morphology.^135^

In the case of Ebstein's anomaly, myocardial deformation imaging has little to contribute to right ventricular function assessment, since strain has a weak correlation with ejection fraction measured with MR.^136^

Castaldi et al.^137^ have demonstrated the utility of left ventricle longitudinal strain to diagnosis of patients with coronary obstruction in late PO after correction of anomalous origin of the left coronary artery. A strain value < –14.8% on echocardiography identified myocardial segments with fibrosis on MR, with sensitivity of 92.5% and specificity of 93.7%.^137^

### 10.3. Right and Left Atrial Strain in Pediatrics

Analysis of right atrial mechanics using speckle tracking was recently introduced in pediatrics, emerging as a promising tool for detection of right ventricular dysfunction. Hope et al.^139^ found a significant reduction in right atrium longitudinal strain in children with IPH. Atrial strain proved more sensitive and specific than conventional right ventricular function assessment parameters for identifying patients with IPH who would later develop unfavorable outcomes (death, pulmonary and/or cardiac transplant).^139^

Several studies have described the clinical implications of left atrial strain measurements using the speckle tracking technique. Left atrium strain in the reservoir phase proved more accurate for estimation of end-diastolic pressure of the LV than classical echocardiographic parameters such as left atrial volume and the E/E' ratio and was also inversely correlated with plasma NT-ProBNP levels.^140^

### 10.4. Prospects for Utilization of Ventricular Strain in the Fetus

Recent studies have suggested that analysis of myocardial deformation can also contribute to evaluation of biventricular systolic and diastolic function in fetuses. For example, Miranda et al. documented reduced early and late diastolic strain rate in the longitudinal axes of RV and LV in fetuses with diabetic mothers. Additionally, they also observed reductions in right ventricle longitudinal systolic peak strain in comparison with normal fetuses of the same gestational age. These authors pointed out that diastolic deformation compromise was irrespective of the presence of septal hypertrophy. They concluded that myocardial deformation analysis could detect subclinical changes in the fetuses of diabetic mothers before classical echocardiographic parameters are able to do so.^141^

Dusenbery et al.^138^ confirmed the association between reduced LV longitudinal strain and presence of myocardial fibrosis, assessing children and young adults with aortic valve stenosis and preserved LV ejection fraction.^138^ It is known that adults with aortic stenosis who have late enhancement on MR with gadolinium and reduced LV longitudinal strain values have higher mortality rates after valve interventions.^138^ See Table 15 for recommendation classes and evidence levels.

**Table 15:** Recommendations for ventricular strain in congenital heart disease^129^^–^^135^^,^^137^

Indication	Recommendation class	Evidence level
Functional evaluation of subpulmonary RV (e.g. T4F)	IIb	B
Functional evaluation of systemic RV (e.g. PO of Senning procedure, CCTGA)	IIb	B
Evaluation of SV before Fontan procedure	IIb	B
Evaluation of SV after Fontan procedure	IIb	B
Assessment of LV after ALCAPA surgical repair	IIa	B
Evaluation of LV function in aortic stenosis	IIb	B

*ALCAPA: anomalous left main coronary artery from the pulmonary artery; CCTGA: congenitally corrected transpositionof thegreat arteries; LV: left ventricle; PO: postoperative; RV: right ventricle; SV: single ventricle; T4F: tetralogy of Fallot.*
